# Triple threat: a review on nanoplastic ecotoxicity, pollutant co-exposures, and climate change in freshwater organisms

**DOI:** 10.3389/fphys.2026.1808330

**Published:** 2026-04-16

**Authors:** Analía Ale, Victoria S. Andrade, Lidwina Bertrand, María Florencia Gutierrez

**Affiliations:** 1 Cátedra de Toxicología, Farmacología y Bioquímica Legal, Facultad de Bioquímica y Ciencias Biológicas, Universidad Nacional del Litoral (FBCB-UNL), CONICET, Santa Fe, Argentina; 2 Instituto Nacional de Limnología (CONICET-UNL), Santa Fe, Argentina; 3 Laboratorio de Investigaciones en Contaminación Acuática y Ecotoxicología (LICAE), Centro de Investigaciones Bioquímicas e Inmunología (CIBICI), Consejo Nacional de Investigaciones Científicas y Técnicas (CONICET), Córdoba, Argentina; 4 Facultad de Ciencias Químicas, Departamento de Bioquímica Clínica, Universidad Nacional de Córdoba (UNC), Córdoba, Argentina; 5 Escuela Superior de Sanidad “Dr. Ramón Carrillo” (FBCB-UNL), Santa Fe, Argentina

**Keywords:** nano-sized plastics, oxidative stress, synergism, temperature, Trojan horse effect

## Abstract

Plastic-waste pollution has become one of the major threats to the entire environment, including the aquatic ecosystems. Vast literature is available on microplastics ecotoxicity; however, further degradation leads to nano-sized plastics, or nanoplastics (NP), which were reported to be more reactive and even more toxic for the aquatic biota despite the fact that they were studied to a lesser extent. In a context of a changing world, where freshwater systems are particularly sensitive, and the ecotoxicology of plastic as a nanopollutant has been poorly addressed in comparison with the marine environment, the objective of this study is to evaluate the physiological effects in case of NP co-exposures with other pollutants and/or stressors, and also provide further insights into in a context of climate change (CC) based on peer-reviewed literature published between 2020 and 2025. The most represented groups were freshwater algae, microinvertebrate and fish; however, they were predominantly represented by a few model species: *Chlorella* spp. alga, *Daphnia magna* microcrustacean, and *Danio rerio* fish, respectively. Metals and pesticides were the most frequently studied co-stressors. Synergistic interactions emerged as particularly relevant, often linked to NP acting as pollutant vectors through Trojan horse-derived mechanism. Regarding CC, rising temperature was the most assessed variable, generally enhancing NP toxicity in freshwater organisms. Our findings highlight the complexity of realistic co-exposure scenarios and emphasize the need for ecotoxicological studies that address multiple stressors in a changing world.

## Graphical Abstract

**Figure:**
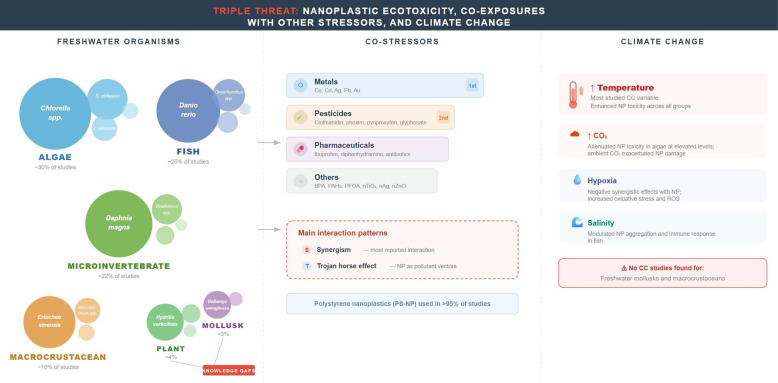

## Highlights

- Nanoplastics (NP) under co-exposure scenarios remain unexplored for freshwater species
- Synergism was the most analyzed interaction mediated by the Trojan horse mechanism
- Rising temperature enhanced NP ecotoxicity across different organisms’ groups
- *Chlorella* spp., *Daphnia magna*, and *Danio rerio* were the main studied species
- Mollusk and macrocrustacean represent critical knowledge gaps under climate change scenarios

## Introduction

Plastic has become an essential part of modern life, with numerous applications in everyday activities. Its production began in the 1950s and had reached 367 million metric tons by 2020 (Narwal and Kakakhel, 2025). In parallel, plastic waste has been considered as an emerging environmental problem since the 70s, and currently, it constitutes one of the most important challenges worldwide given the need for providing solutions and suitable management and further decrease in plastic-based consumption and disposal. In this regard, only in the last five decades plastic production has grown at an annual rate of 8.7%, resulting in global production that exceeds 400 million tons (Arif et al., 2024; Ng et al., 2018; Plastic Europe, 2024). In particular, freshwater ecosystems are increasingly threatened by plastic-based pollution, including nanoplastic (NP); a product of the continued degradation of larger plastic debris, whose ecotoxicological effects remain poorly understood compared to their micrometric counterparts. Furthermore, under realistic environmental scenarios, NP co-occur with other pollutants and are subject to climate change (CC)-derived stressors, yet the combined effects of these multiple stressors on freshwater organisms have been scarcely addressed. This knowledge gap constitutes a major concern for environmental risk assessment and forms the main objective and basis for the present review.

Plastic particles have been classified into two categories according to their sources: primary and secondary. The first are originally manufactured according to the desirable size, meanwhile secondary particles originate from fragmentation processes of larger plastic debris (Gigault et al., 2016). Plastic reaches environmental matrices through multiple pathways, including direct discharge from municipal and industrial wastewater as well as generation via environmental degradation (physical, chemical or biological) of larger plastic items (Bashir et al., 2021). Through progressive fragmentation, these particles can reach the micro- and nanoscale, ultimately forming NP. In this context, microplastic has been defined as those particles ≤ 5 mm in size and > 1 µm (Kaur et al., 2023), and NP refers to those particles in homo and heteroaggregates within a size ranging from 1 to 1000 nm, thus often exhibit a colloidal behavior (Arif et al., 2024; Dhada et al., 2023). Here it should be noted that a consensus on NP size definition has not yet been reached, as some authors adopt a narrower range of 1–100 nm, consistent with the classical definition of nanomaterials (Hartmann et al., 2019). However, in the present study we adopted the 1–1000 nm threshold following the most widely used definition in ecotoxicological NP-based research (Gigault et al., 2018).

Specifically, when compared to microplastics, for which toxicity has been widely reported, NP are more reactive and harmful despite that their environmental processes are less understood, bringing a complex environmental concern (Lee and Fang, 2022). Interestingly, it has been discussed that NP are likely to become more prevalent in the future due to both the increased demand and production of engineered nano-based products industrial applications, and the continued degradation of the already existing (micro)plastics in the environments (Lee and Fang, 2022). Given their nanometric size, NP are more prone to translocate into tissues and enter cells than their micrometric counterparts. However, in aquatic environments, their impact on the biota is regulated by their behavior when they aggregate, which depends on the colloidal stability. Such a phenomenon can modify exposure conditions and ultimate risk for organisms inhabiting the water column or sediment (Redondo-Hasselerharm et al., 2020).

Despite receiving large volumes of plastic waste, freshwater systems have been studied to a lesser extent than marine environments in terms of related pollution. This is particularly important in the case of rivers, which are major recipients of discharges from urban areas. Furthermore, this issue is often exacerbated by the absence of adequate wastewater treatment plants and improper waste management practices (Jaikumar et al., 2025). Although NP ecotoxicity research is still in its early stages compared to microplastics, single-exposure studies have demonstrated a range of adverse effects across freshwater taxonomic groups. In algae, NP have been shown to inhibit growth, impair photosynthesis, and induce oxidative stress (Reichelt and Gorokhova, 2020). In microinvertebrates, particularly *D. magna*, documented effects include immobilization, reproductive impairment, and altered swimming behavior (Yin et al., 2023). In fish, developmental abnormalities, neurotoxicity, and metabolic disruption have been reported, especially in *D. rerio* embryo and larvae (Barreto et al., 2023a). However, in natural freshwater systems, organisms are rarely exposed to NP in isolation.

The lack of knowledge in terms of plastic-based nanotoxicity in freshwater biota raises concern given the complexity of NP behavior in environmental matrices and their poorly addressed environmental fate. Importantly, NP were well established as vectors, because of their capacity to transport other substances, like metals and organic ones (Oger et al., 2025). Accordingly, the mechanism called “Trojan horse” effect, also applied for other kinds of nanomaterials, was discussed through the current bibliography as NP act as vehicles of co-existing chemicals. This phenomenon has been explained by the enhanced particle penetration inside the aquatic organism’s tissues, increasing their bioavailability, internalization, and even exerting additional toxicity mechanisms which will depend on particle’s intrinsic properties and further environmental factors (Zhang and Xu, 2022).

Global CC has been considered as a major threat to aquatic ecosystems. Freshwater environments are particularly vulnerable since they are threatened by a range of anthropogenic stressors, including the derived from CC. In this context, those stressors could have an additive effect, thus their individual effect may be underestimated (Gutierrez et al., 2025). According to the Intergovernmental Panel on Climate Change (IPCC), raising temperatures are projected in line with greenhouse gas emissions. Alongside global warming, CC will also induce shifts in precipitation and evaporation patterns, resulting in substantial changes to the physicochemical conditions of freshwater systems (IPCC, 2023). Having said that, there is a need for elucidating the potential responses in the associated biota facing a world challenged with a triple threat. This should include research on NP interacting with co-occurring pollutants NP, as well as factors representing CC scenarios, and further nanotoxicological interactions among the implicated stressors. While the ecotoxicity and health risks of micro- and nanoplastics have been broadly recognized (WHO, 2022; Ansari et al., 2025), the specific interactions arising from NP co-exposure with other environmental pollutants under CC scenarios in freshwater systems remain unaddressed, which constitutes the central gap this review aims to fill. In the context of a changing world and the need for a better understanding of the nanotoxicity of plastics in freshwater organisms, this study aims to evaluate the physiological effects in case of NP co-exposures with other pollutants and/or stressors in freshwater organisms, and also provide further insights in a context of CC.

## Bibliographic data acquisition

Exhaustive literature research was conducted limited to research papers that employed experimental designs involving waterborne exposure and aquatic test organisms with a complete life cycle in freshwater. The following datasets were employed: Scopus (https://www.scopus.com), Google Scholar (https://scholar.google.com), PubMed (https://pubmed.ncbi.nlm.nih.gov), ScienceDirect (https://www.sciencedirect.com), and Scielo (https://scielo.org). Only peer-reviewed research articles published between January 2020, and December 2025 were considered. The employed keywords (and their combinations) were: “nanoplastics”, “interaction”, “microorganisms”, “co-exposure”, “co-occurrence”, “organic matter”, “pH”, “temperature”, “toxicity”, “salinity”, “vectors”, “fish”, “algae”, “crustacean”, “microinvertebrates”, “microcrustacean”, “macrocrustacean”, “aquatic organisms”, “aquatic plants”, and “climate change”. Neither studies based on single NP exposures nor mesocosms-based were included in this study. No other language than English was considered. The criteria adopted for “nanoplastic” definition was plastic-based particles ≤ 1 µm, and for “climate-change derived stressors” included different temperatures, salinities, dissolved organic matter levels, hypoxia levels, and CO_2_ levels. A detailed flow diagram showing the criteria adopted for selecting the studies is provided in **Supplementary Figure 1** (**Supplementary Material**).

## Results and discussion

**Figure 1:**
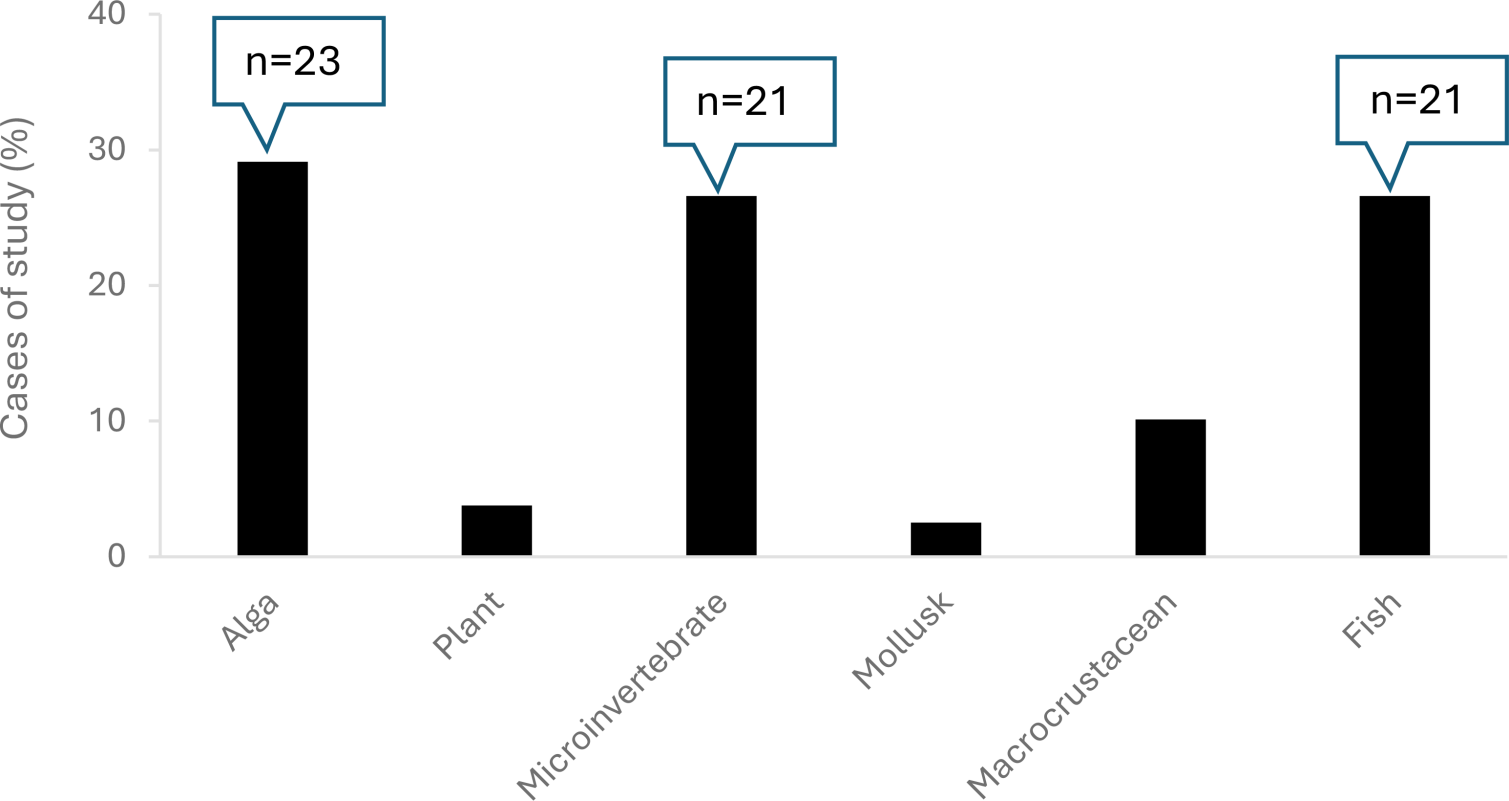
Percentages of studies per organism group.

**Figure 2:**
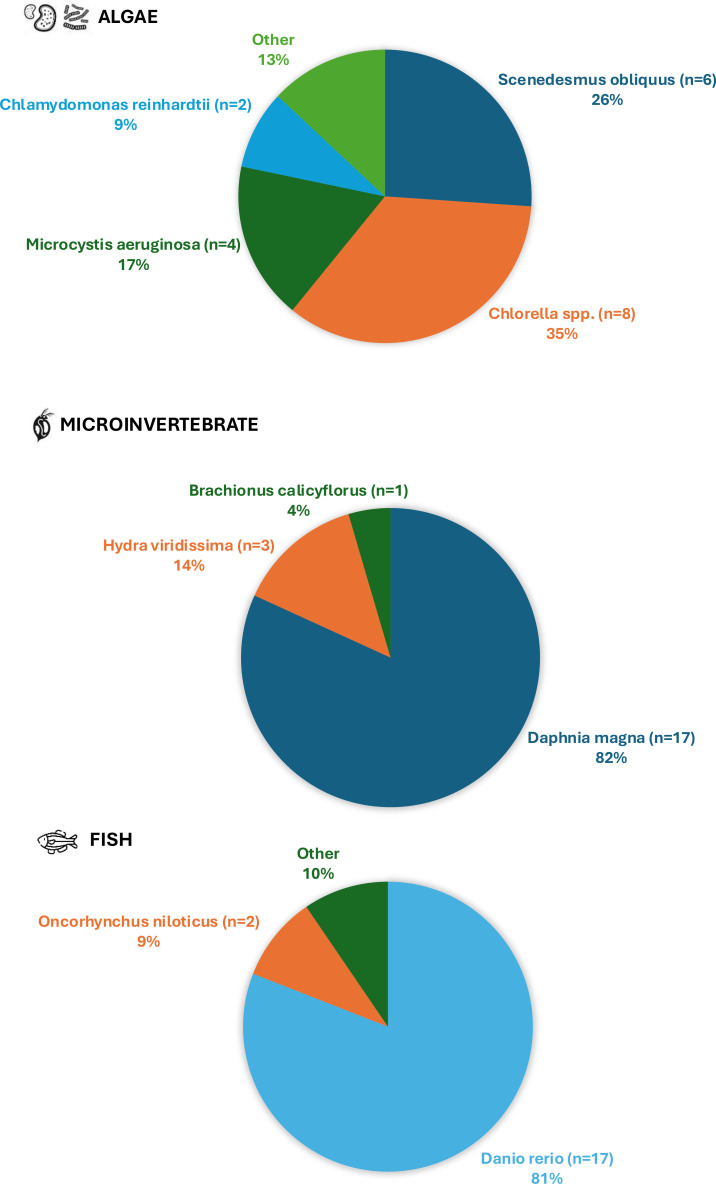
Most represented species in the top three groups: algae, microinvertebrate, and fish.

Based on our research criteria, we obtained a total of 78 cases of studies represented by a total of 58 peer-reviewed articles, which are shown and summarized in **Supplementary Table 1** (**Supplementary Material**) according to their biological group: algae, plants, microinvertebrates, macrocrustaceans, mollusks, and fish. In this context, **Figure 1** shows the group of organisms that have been studied the most, with freshwater algae, microinvertebrates and fish being the top three. However, low taxonomic diversity was observed in the studies analyzed, with the most commonly studied species being *Chlorella* spp. for algae, *Daphnia magna* for microcrustacean, and *Danio rerio* for fish (**Figure 2**).

**Figure 3:**
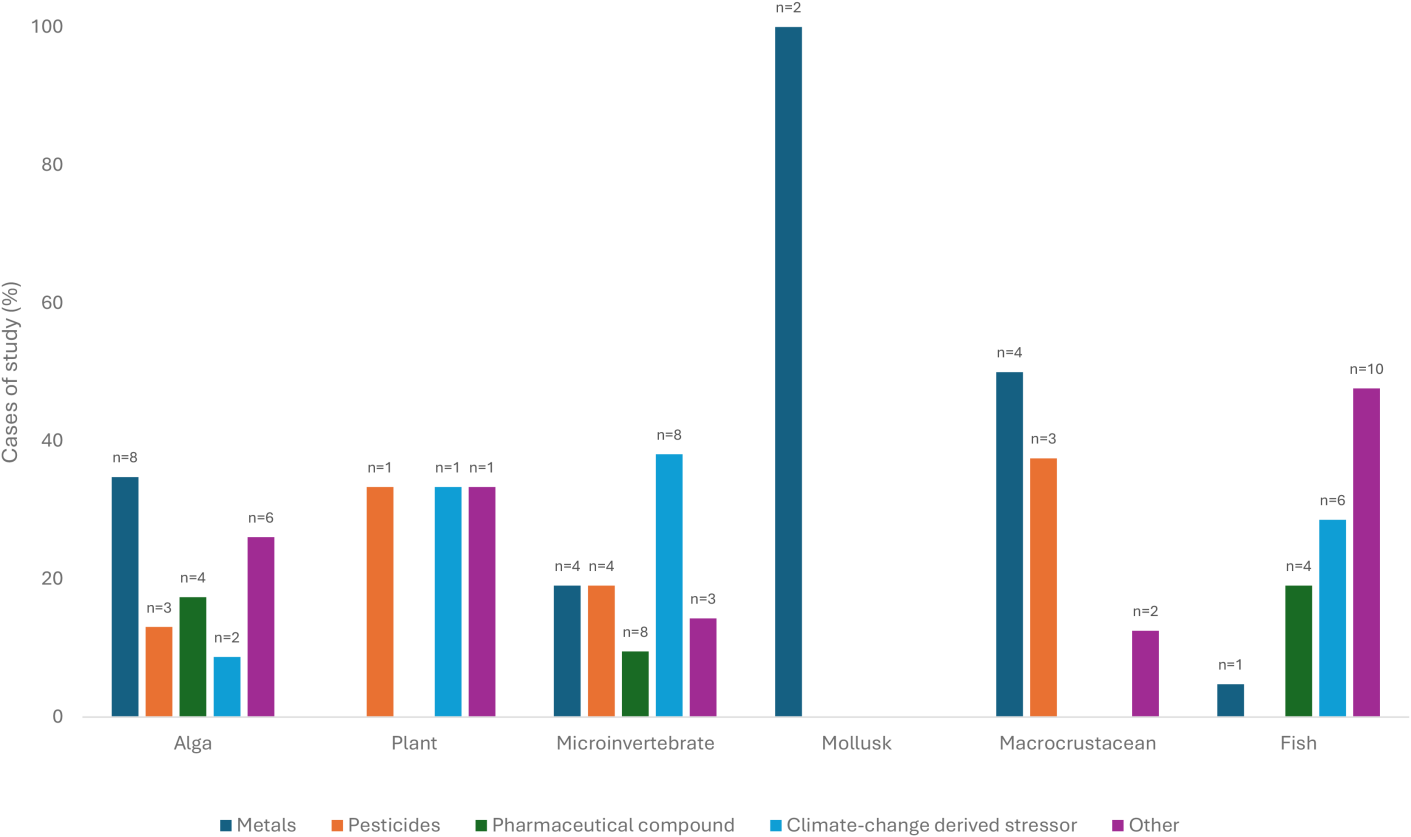
Percentages of studies for each group of organisms considering co-exposure NP to alongside different stressor categories.

On the other hand, **Figure 3** shows that the most commonly studied stressors under co-exposure to NP are metal, pesticide, pharmaceutical compound, and CC conditions, which vary according to the organism group considered. For algae and macrocrustacean, the most studied co-exposure stressor were metals. While each of the three available studies on the plant group are based on different kinds of stressors, the only two cases for mollusk are represented by metals as co-pollutants. Interestingly, most of the studies carried out on microinvertebrate and fish involved co-exposure to other kinds of stressors, such as polycyclic aromatic hydrocarbons (please see **Supplementary Table 1** for further details).

On the other hand, a total of 21 countries were involved in conducting the selected studies. Notably, China was the most represented country (~50% of studies), followed by Canada, Brazil, and India (**Supplementary Table 2**, **Supplementary Material**). This geographic imbalance further underscores the need for broader representation, particularly from regions with high freshwater biodiversity that (e.g., South America, sub-Saharan Africa, and Southeast Asia) that remain underexplored.

**Figure 4:**
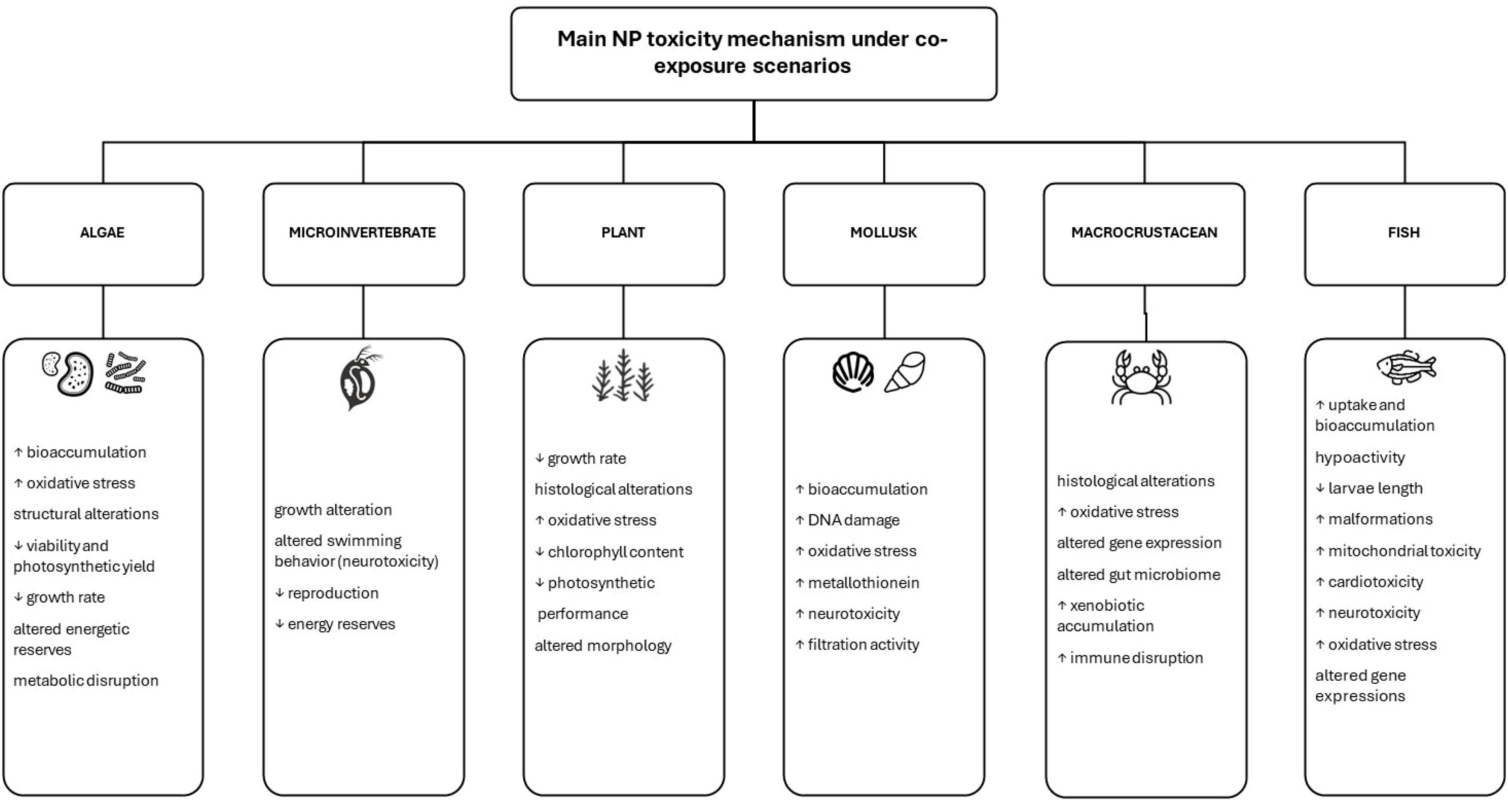
Main toxicity mechanisms across the freshwater organism’s groups in case of co-exposure to NP and other pollutants/stressors.

The following discussion presents the main up-to-date findings on NP pollution and co-exposure to other pollutants and/or stressors, as well as the implications of CC derived stressors for freshwater organisms. Finally, **Figure 4** summarizes the main toxicity mechanisms studied within the different taxonomic groups.

### Algae

Among the Chlorophyta taxa, *Chlorella vulgaris* and *Scenedesmus obliquus* were the most frequently investigated species. Cyanobacteria were less represented, with *Microcystis aeruginosa* being the dominant model species. Polystyrene (PS) was the only polymer used in all the studies for tested NP (PS-NP), with particle sizes ranging from 40 to 800 nm.

In chlorophytes, co-exposure to NP and metals generally suggests that particles can act as carriers and/or modulate toxicity through synergistic interactions. In *Euglena gracilis*, co-exposure to NP and Cd resulted in synergistic growth inhibition accompanied by activated antioxidant defense responses and disruption of carbohydrate and purine metabolism (Cao et al., 2022). NP enhanced Cu toxicity in *Chlorella* sp. and *Pseudokirchneriella subcapitata* by inducing oxidative stress and morphological alterations (Wan et al., 2021), while Pb co-exposure caused synergistic effects leading to reduced cell size and increased aggregation (Khoshnamvand et al., 2024). In *Scenedesmus* sp., NP intensified Cd toxicity by altering cell wall polysaccharide composition, while high Cd concentrations facilitated NP internalization by loosening cell wall structure (Zhang et al., 2024).

The interaction between nanometals and NP further highlighted complex response patterns. Co-exposure to titanium dioxide nanoparticles (nTiO_2_) and NP decreased cell viability and induced oxidative stress in *S. obliquus* and *Scenedesmus* sp. Additionally, combined exposure to nTiO_2_ and fluorescent NP altered algal morphology and reduced photosynthetic yield and esterase activity (Das et al., 2022). In contrast, antagonistic interactions were reported for NP and Ag nanoparticles (nAg) in *Chlorella* sp., where PS-NP reduced nAg toxicity by adsorbing Ag and promoting particle aggregation, thereby lowering nAg bioavailability (Li et al., 2025). Furthermore, Li et al. (2024) showed that biochar mitigated the combined toxicity of NP and silver nanoparticles (nAg) toward *C. vulgaris*, suggesting that natural carbonaceous materials may modulate NP-nanometal interactions in freshwater systems.

Organic pollutants exhibited more variable interaction patterns. For pesticides, both antagonistic and synergistic effects have been documented. The insecticide clothianidin showed antagonistic interactions with PS-NP in *Chlamydomonas reinhardtii* based on growth inhibition assays (Bandeira et al., 2025). Conversely, the herbicide atrazine displayed synergistic toxicity when combined with NP, negatively affecting biomass, chlorophyll *a* and *b*, antioxidant capacity, protein content, and oxidative stress biomarkers. These effects were attributed to increased atrazine bioavailability mediated by NP-algae interactions (Khoshnamvand et al., 2024).

Pharmaceutical compounds generally exhibited antagonistic or mitigative interactions with NP. For example, ibuprofen (IBU) caused less growth inhibition in *Chlorella pyrenoidosa* when combined with PS-NP compared to single-compound exposures. This response was linked to reduced bioaccumulation and enhanced biodegradation of IBU in the presence of NP, resulting in increased removal from the medium (Wang et al., 2020). Diclofenac showed dose-dependent interactions, characterized by antagonistic effects at low concentrations and synergistic toxicity at higher concentrations. This was reflected in oxidative stress parameters, antioxidant enzyme activities, and photosynthetic pigment content (Christudoss et al., 2024).

Similarly, the toxicity of sulfonamide antibiotics (sulfapyridine, sulfamethazine, sulfamethoxypyridazine, and sulfamethoxazole) with PS-NP co-exposure was generally lower than that of the individual compounds in *S. obliquus*, indicating antagonistic interactions (Yang et al., 2025). In *C. reinhardtii*, joint exposure to NP and sulfamethoxazole under light conditions resulted in a strong mitigative effect, likely due to enhanced adsorption of the antibiotic onto NP, reducing its bioavailability in exposure media (Wang et al., 2023a).

Moreover, natural organic matter and algal exudates, particularly extracellular polymeric substances (EPS), frequently mitigate NP toxicity. In *S. obliquus*, co-exposure to EPS and NP reduced reactive oxygen species production, lowered antioxidant enzyme activity (superoxide dismutase, SOD, and catalase, CAT), and increased cell viability, ultimately improving photosynthetic efficiency compared to NP exposure alone (Giri and Mukherjee, 2021). Likewise, humic acids significantly alleviated NP-induced reductions in biomass and chlorophyll *a* content in *C. vulgaris* (Hanachi et al., 2022).

Climate-related stressors also modulated the effects of PS-NP in chlorophytes. Low temperature and ambient CO_2_ conditions exacerbated NP-induced damage, whereas elevated CO_2_ concentrations and warmer temperatures attenuated NP toxicity in *S. obliquus*, highlighting the importance of considering future climate scenarios when predicting the impact of NP (Yang et al., 2020).

In cyanobacteria, the combined effects of PS-NP and other stressors remain comparatively understudied. In *M. aeruginosa*, co-exposure to Cd and NP (80 nm-sized) at concentrations between 0.5 and 5 mg/L resulted in synergistic effects, including increased microcystin-LR production and alterations in chlorophyll a content and enzymatic activity (Wang et al., 2023b). In contrast, the coexistence of PS-NP and CuSO_4_ exacerbated Cu-induced impairment of photosynthetic activity and metabolic processes but reduced microcystin-LR concentrations in the water column (Mamatimin et al., 2025).

Several studies reported antagonistic interactions in cyanobacteria. Clothianidin toxicity to *M. aeruginosa* was reduced in the presence of NP (Bandeira et al., 2025), and PS-NP weakened the inhibitory effects of H_2_O_2_ on cell abundance and microcystin production (Guo et al., 2021). Similarly, polybrominated diphenyl ethers (PBDE) showed reduced toxicity to Arctic cyanobacteria when co-occurring with NP, attributed to aggregation and PBDE adsorption (Xin et al., 2022). This contrasts with findings in S. obliquus, where co-exposure to NP and tetrabromobisphenol A (TBBPA) enhanced growth inhibition, oxidative stress, and reduced photosynthetic pigments relative to TBBPA alone (Xin et al., 2022).

From a mechanistic perspective at the subcellular level, NP combined with metals induced ultrastructural changes in cell organelles, including alterations to chloroplast integrity and vacuole formation (Wan et al., 2021). Cell wall composition was also modified, particularly polysaccharide content, which in turn facilitated NP internalization under high metal concentrations (Zhang et al., 2024). Metabolic disruption was evidenced through alterations in carbohydrate and purine metabolism-related pathways under NP-Cd co-exposure (Cao et al., 2022), while co-exposure with the algaecide CuSO_4_ altered broader metabolic processes in *M. aeruginosa* (Mamatimin et al., 2025). At the molecular level, co-exposure to NP and sulfonamide antibiotics affected physiological and biochemical parameters linked to gene expression regulation in *S. obliquus* (Yang et al., 2025). However, effects on cell-cell interactions and nutrient uptake under co-exposure scenarios remain unexplored, representing an important gap for future research.

### Microinvertebrate

In recent literature, a total of ten reports studied the combined effects of NP and pollutants in microcrustaceans, all focused on PS-NP effects on *Daphnia magna*, with pesticides being the main co-stressors. In general, pesticide co-exposure resulted predominantly in antagonistic interactions. Bandeira et al. (2025) reported that NP caused mortality, growth, reproduction, and swimming alterations in *D. magna* co-exposed to clothianidin, with antagonism as the predominant interaction. Similarly, Jia et al. (2024) observed that NP reduced pyriproxyfen toxicity on survival, reproduction, and growth, while also decreasing NP uptake. Zhang et al. (2024) showed that NP reduced chlorpyrifos lethality, likely due to insecticide adsorption onto particles followed by aggregation and sedimentation. In contrast, Nogueira et al. (2022) reported that NP and glyphosate co-occurrence increased toxicity in terms of mortality, reactive oxygen species (ROS) production, swimming behavior, and reproduction across F1 and F2 generations, representing the only case of synergistic interaction with a pesticide in this group. Collectively, these studies indicate that NP-pesticide interactions in *D. magna* primarily modulate survival, growth, reproduction, and swimming behavior, with antagonistic effects predominating, likely driven by NP adsorption and reduced pesticide bioavailability.

Regarding pharmaceutical compounds, only two studies reported their interactive effects on microcrustaceans. Barreto et al. (2023b) observed synergistic interactions between PS-NP and diphenhydramine on *D. magna*, evidenced by increased oxidative stress biomarkers and disruption of energy metabolism-related genes. Maszczyk et al. (2022) found that co-exposure to NP and enrofloxacin resulted in negative interactions, with increased toxicity in terms of body volume, clutch size, egg volume, and gut microbiota respiration rate.

Few reports analyzed the effects of PS-NP in combination with other pollutants. Sun et al. (2024) observed that NP and halogenated PAH (polycyclic aromatic hydrocarbons) co-exposure in *D. magna* resulted in antagonistic or additive effects on mortality, depending on NP size, with smaller particles showing enhanced toxicity. Wang et al. (2024) reported that NP, in triple mixtures with carbamazepine and fulvic acid, enhanced antibiotic bioaccumulation in *D. magna*. Pashaei et al. (2023) observed that NP co-exposure with triclosan increased *D. magna* mortality, while the combination with caffeine showed no clear interaction pattern.

Beyond *D. magna*, only limited studies examined NP combined effects in other freshwater microinvertebrates. Saavedra et al. (2019) showed that NP surface charge influenced eco-corona formation and aggregation, modulating toxicity to the rotifer *Brachionus calyciflorus*. Santos et al. (2024) evaluated NP co-exposure with Cd, Zn, and Cu in *Hydra viridissima*, finding metal-specific responses: Cd and Zn toxicity was enhanced by NP, whereas Cu toxicity remained unaffected. These findings highlight the importance of metal-specific chemistry and NP-metal interactions in determining biological outcomes.

Unlike anthropogenic chemicals, natural components such as dissolved organic matter can reduce PS-NP toxicity through the formation of eco-coronas. However, the physicochemical properties of these eco-coronas, particularly surface charge, strongly influence biological responses. In this regard, organic matter significantly mitigated the toxicity of amidine- and carboxyl-functionalized NP in the rotifer *Brachionus calyciflorus* (Saavedra et al., 2019), underscoring the protective role of natural organic matter.

From a climate-change (CC) perspective, only three reports analyzed the incidence of temperature on PS-NP effects in *D. magna*. Chang et al. (2023) reported that NP effects were apparent only under warming conditions (+4 °C) and daily temperature fluctuations, with increased fecundity, heat tolerance, and CYP450 activity. Xu et al. (2024) found that NP decreased heart rate, thoracic limb activity, and feeding rate, with effects enhanced by temperature increase and daily fluctuations. Sanpradit et al. (2024) observed that a 5 °C increase augmented NP lethality approximately fourfold, while NP accumulation decreased due to enhanced elimination, reproduction declined, and oxidative stress increased. Collectively, these studies indicate that rising temperatures consistently exacerbate NP toxicity in microcrustaceans through enhanced bioavailability, metabolic disruption, and oxidative stress. Other reports analyzed the effects of NP in combination with variables that also could be related to the consequences of CC. Lee et al. (2024) reported that the combined exposure of *D. magna* to PS-NP and hypoxia resulted in negative synergic interactions, as they observed increased mortality, oxidative stress, and decreased reproduction and growth. On the other hand, Fadare et al. (2020) reported that the presence of natural organic matter decreased the effects of PS-NP on *D. magna* in terms of lethality, and upregulation of genes. They observed that organic matter was adsorbed on NP and changed the distribution of the particles in *D. magna* neonates, leading to alleviated toxicity. Overall, co-exposure studies in microinvertebrates reveal that NP interactions affect multiple mechanistic endpoints, including oxidative stress and antioxidant defense activation, reproductive impairment, altered swimming behavior and feeding rate, and neurotoxicity-related responses such as acetylcholinesterase inhibition. Although data remains scarce, existing studies demonstrate that NP interactions in microinvertebrates are highly context-dependent and mediated by species traits, contaminant type, and environmental chemistry. These findings emphasize the need for broader taxonomic coverage and for experiments incorporating environmentally relevant mixtures and natural modifiers.

### Plant

Information on the combined effects of PS-NP and other stressors on aquatic macrophytes remains extremely limited (three studies found). Nevertheless, the available evidence suggests that both anthropogenic contaminants and natural environmental factors can substantially modulate NP toxicity in these primary producers through multiple physiological and biochemical pathways.

In *Hydrilla verticillata*, co-exposure to bisphenol F (BPF) and NP resulted in reduced growth rates and chlorophyll content. This response was accompanied by pronounced oxidative stress, as indicated by increased enzyme activities, apart from elevated malondialdehyde (MDA) levels. Despite these physiological impairments, the joint exposure of NP and BPF produced an antagonistic interaction with respect to plant growth. Mechanistically, BPF enhanced the adsorption of NP onto leaf surfaces, potentially limiting NP internalization and partially mitigating growth inhibition (Yu et al., 2022).

In floating macrophytes, evidence is similarly scarce. In *Lemna minor*, co-exposure to PS-NP and the neonicotinoid insecticide clothianidin resulted in interaction patterns ranging from antagonistic to additive, based on growth inhibition assays (Bandeira et al., 2025). These findings indicate that NP may modulate pesticide bioavailability and uptake in small aquatic plants, although the underlying mechanisms remain largely unresolved.

Stressors associated with CC also appear to influence NP effects in macrophytes. In the aquatic fern *Azolla filiculoides*, combined exposure to elevated temperature and PS-NP exacerbated adverse effects on histological integrity, morphology, and photosynthetic performance (Bottega et al., 2024). These effects were consistent with an increased uptake of NP under warmer conditions, suggesting that rising temperatures may enhance NP-plant interactions and toxicity.

Overall, the limited number of studies available highlights significant knowledge gaps regarding NP mixtures in aquatic macrophytes. Current evidence suggests that interactions may range from antagonistic to stress-amplifying, depending on the co-occurring contaminant or environmental factor, plant morphology, and uptake pathways. Given the ecological importance of macrophytes in nutrient cycling, habitat structuring, and contaminant retention in freshwater systems, further research addressing environmentally realistic mixtures and climate-related stressors is urgently needed.

### Mollusk

Only two studies met our research criteria for freshwater mollusks, representing a significant knowledge gap. Despite being poorly studied, the sensitivity of this group to nanopollutants has been highlighted, especially for filter-feeding species (Ale et al., 2025; Skawina et al., 2024). Luo et al. (2022) exposed the snail *Bellamya aeruginosa* to PS-NP and Cd for 28 days, showing that NP significantly increased metal bioavailability, facilitating uptake and bioaccumulation. Greater DNA damage and oxidative stress were evidenced under co-exposure, with reduced metallothionein expression suggesting synergistic toxic effects. Arini et al. (2023) exposed the bivalve *Corbicula fluminea* to PS-NP and AlCl_3_ via both waterborne and dietary routes for 21 days, revealing synergistic toxicity through trophic exposure in terms of oxidative stress and neurotoxicity. Increased filtration activity suggested that NP affected both neurotoxicity and xenobiotic accumulation capacities. Importantly, the authors highlighted the relevance of using naturally aged NP rather than synthetic latexes. From a mechanistic perspective, the available evidence for mollusks points to oxidative stress, DNA damage, metallothionein suppression, neurotoxicity, and altered filtration activity as key responses under co-exposure conditions.

### Macrocrustacean

A total of eight experimental studies assessed NP effects on freshwater macrocrustaceans, all employing PS-NP of approximately 100 nm in decapod species. The crab *Eriocheir sinensis* was the most frequently studied species (seven studies), with only one report on *Macrobrachium rosenbergii*.

Regarding the co-exposure of NP with metals, four studies were conducted. Xu et al. (2025a, b) investigated PS-NP and copper effects on *Eriocheir sinensis* over 21 days, revealing tissue-specific responses. In gills, co-exposure enhanced antioxidant enzyme activity and disrupted energy metabolism, with suppressed immunity and detoxification gene expression (Xu et al., 2025a). In hepatopancreas, time-dependent NP and Cu accumulation was observed, although Cu bioavailability appeared reduced in the presence of NP. Transcriptomic analyses indicated disrupted lipid metabolism and activation of compensatory immune mechanisms under co-exposure (Xu et al., 2025b). In some cases co-exposure reversed the toxic effects of individual treatments, suggesting complex non-additive interactions. Similarly, Che et al. (2024a) reported pronounced but non-additive intestinal responses to PS-NP and Cd in *E. sinensis*, including oxidative stress, disrupted microbiota homeostasis, and disturbances in pathways associated with ferroptosis, apoptosis, and immune dysfunction. Again, co-exposure partially mitigated intestinal toxicity relative to single exposures. Only one study assessed multi-metal mixtures, exposing *M. rosenbergii* to PS-NP and a heavy metal cocktail (Banaee et al., 2025). Co-exposure induced pronounced oxidative stress, disrupted metal homeostasis, impaired metabolism, and reduced survival. NP significantly increased metal bioaccumulation in shrimp tissues, supporting a carrier role for NP in metal uptake, accompanied by extensive cellular injury including protein oxidation and membrane damage.

Of the four studies on NP co-exposure with organic xenobiotics, three assessed the pesticide phoxim (PHO) in *E. sinensis*. Che et al. (2024b) reported intestinal toxicity characterized by histopathological alterations, oxidative stress, inflammatory gene activation, and disrupted microbiota. However, co-exposure partially mitigated intestinal inflammatory responses compared to single exposures. Ding et al. (2025) confirmed intestinal histological abnormalities, but interestingly, while individual exposures thinned the peritrophic membrane, co-exposure increased its thickness. Individual exposures also increased pathogenic bacteria abundance, whereas co-exposure reduced them, suggesting complex modulatory effects. Huang et al. (2024) focused on hepatopancreatic effects, showing that co-exposure exacerbated inflammation through activation of the NF-κB signaling pathway, despite individual exposures suggesting inflammatory recession. Collectively, these studies highlight that NP-PHO co-exposure in *E. sinensis* elicits non-additive effects, mitigating certain intestinal inflammatory responses while aggravating systemic and hepatopancreatic toxicity.

Finally, Huang et al. (2025) evaluated PS-NP and PFOA co-exposure in *E. sinensis* over 28 days, reporting amplified hepatopancreatic oxidative damage, disrupted lipid metabolism, and intestinal inflammatory responses through proliferation of pathogenic bacteria, reinforcing the role of NP as effect modifiers that aggravate contaminant-induced toxicity. Notably, no publications were identified that considered NP interactions under exposure scenarios associated with CC-related stressors, highlighting another critical knowledge gap regarding how multiple environmentally relevant factors may jointly influence NP toxicity in freshwater macrocrustaceans.

### Fish

According to our results, *Danio rerio* was the most studied species for assessing PS-NP toxicity under co-exposure conditions. Barreto et al. (2023a) compiled eleven previous cases of study in this species, and our research added another ten, which also included other fish species although represented by a minority.

Among the studies compiled by Barreto et al. (2023a), co-exposure to NP and 17α-ethynylestradiol induced hypoactivity and reduced body larvae length in zebrafish embryo (Chen et al., 2017a), while NP combined with bisphenol A increased particle uptake and accumulation in various tissues of adult fish (Chen et al., 2017b). Lee et al. (2019) reported that smaller NP were more rapidly internalized, and synergistic effects with chloroauric acid were evidenced in a dose-dependent manner for malformations, cell death, and ROS generation. Regarding polycyclic aromatic hydrocarbons (PAH), Trevisan et al. (2019) observed decreased NP uptake due to sorbing, while Trevisan et al. (2020) demonstrated that NP mediated PAH transfer to specific organs, increasing mitochondrial toxicity and potentially affecting the nervous system. Co-exposure to NP and phenanthrene induced more pronounced malformations, increased heartbeat rate, and shortened body length compared to single exposures (Xu et al., 2021). For pharmaceuticals, simvastatin co-occurrence with NP resulted in less pronounced sublethal effects than single-compound exposures (Barreto et al., 2021), whereas the herbicide phenmedipham combined with NP generated hyperactivity and more pronounced biochemical alterations (Santos et al., 2022). Triclosan-NP complexes produced additive, synergistic, or antagonistic behavioral effects depending on particle size (Parenti et al., 2021). Co-exposure to 3,3′,4,4′-tetrachlorobiphenyl (PCB77, a persistent organic pollutant used in electrical equipment) and NP aggravated particle accumulation in a concentration-dependent manner (Li et al., 2022). Lastly, combined exposure to butyl methoxydibenzoylmethane and NP induced neurotoxicity, although single exposures were more severe (Liu et al., 2021).

Based on the additional studies found through our research, Yan et al. (2023) reported that NP acted as carriers for nAg, enhancing metabolic capability while suppressing genotoxicity and lowering apoptosis and immunotoxicity responses. Chronic co-exposure to NP and methylmercury showed that particles redirected metal accumulation toward the head and eyes of fish, intensifying neurotoxicity and increasing mortality (Oger et al., 2025).

In the context of CC, four studies assessed temperature effects on PS-NP toxicity in zebrafish. Duan et al. (2023) observed that elevated temperature promoted NP accumulation in developing fish and enhanced oxidative stress and mitochondrial phosphorylation, showing additive effects; however, omic analyses revealed that warming reduced NP cardiovascular toxicity by enhancing myocardial contractility. Şenol et al. (2023) exposed adult *D. rerio* to NP at 28–30 °C, evidencing DNA damage, liver degeneration, necrosis, and gill inflammation, supported by metabolomic changes indicating protein and lipid oxidation. Similarly, Sulukan et al. (2022) demonstrated deleterious effects on circadian rhythm, brain damage, and metabolite pathway alterations under identical conditions. Trevisan et al. (2025) found that the highest temperature increased developmental markers, apoptosis, and oxygen consumption rates in zebrafish embryo, related to adaptive responses. Notably, under NP exposure, particles seemed to mitigate temperature-driven heart rate changes, although increased oxidative stress and decreased coupling efficiency were registered.

Three studies employed *Oncorhynchus* spp. as test species. Zhang and Goss (2021) exposed *O. mykiss* fingerlings to NP of different sizes combined with PAH and/or humic acids (HA), finding that smaller particles induced greater 7-ethoxyresorufin O-deethylase (EROD) activity in gills and liver, while HA reduced both PAH sorption and EROD activity, suggesting a mitigation effect. The remaining two studies used *O. niloticus* adults and addressed CC-derived stressors. Iqbal et al. (2025) reported that increasing salinity levels exacerbated NP-induced cellular degeneration, necrosis, immune activation, and oxidative damage, identifying salinity as an NP toxicity modulator. Soliman et al. (2025) showed a temperature-dependent increase in biochemical and oxidative stress markers following co-exposure to PVC-NP and elevated temperatures. Lastly, one study was conducted with *Clarias gariepinus* juveniles under different sized PS-NP exposure and antibiotic potassium clavulanate for 15 days, and results showed that the co-exposure with the smallest NP generated the strongest cell apoptosis, DNA damage, and decreases in hematological parameters, together with more pronounced histological effects in spleen. Overall, the authors reported a significant size-dependent interaction for both cytotoxicity and genotoxicity markers (Kamel et al., 2025). Collectively, these results demonstrate that NP toxicity in fish is not only particle size-dependent but also modulated by environmental variables such as salinity and temperature, highlighting the urgent need for climate-integrated risk assessment frameworks.

## Nanoplastic and the triple threat

The concept of “triple threat” refers to the simultaneous exposure of freshwater organisms to nanoplastics (NP), co-occurring pollutants, and climate change (CC)-derived stressors. This realistic scenario reflects actual environmental conditions, where multiple stressors interact in complex ways that cannot be predicted from single-exposure studies. Our review identified two main mechanisms underlying these interactions: the modulation of NP toxicity by CC-derived variables, and the Trojan Horse effect, whereby NP act as vectors facilitating the uptake and bioaccumulation of other contaminants.

### NP and climate change-derived stressors

The interaction between NP and climate change-derived stressors represents a critical and severely underexplored research frontier. This knowledge gap is particularly concerning given that freshwater ecosystems are increasingly vulnerable to simultaneous exposure to multiple stressors.

In algae, CC-related variables showed bidirectional effects on NP toxicity. Yang et al. (2020) demonstrated that elevated temperature and CO_2_ concentrations attenuated NP toxicity in *Scenedesmus obliquus*, whereas environmental conditions exacerbated NP-induced damage through enhanced cellular oxidative stress and impaired photosynthetic capacity. This finding suggests that future climate scenarios may differentially modulate NP impacts on primary producers depending on the specific combination of warming and atmospheric CO_2_ changes, potentially creating regional variation in ecosystem responses.

For microinvertebrates, temperature emerged as the most consistently studied CC-derived stressor. Research assessing temperature effects on *Daphnia magna* demonstrated that elevated temperature exacerbates NP toxicity through multiple mechanistic pathways (Chang et al., 2023; Sanpradit et al., 2024; Xu et al., 2024). Remarkably, Sanpradit et al. (2024) observed a four-fold decrease in LC_50_ values when temperature increased by only 5 °C; a finding with profound implications for predicting NP hazards in warming freshwater systems. Temperature-driven enhancement of NP toxicity appears mediated by increased metabolic rates, enhanced particle uptake, and amplified oxidative stress biomarkers. Beyond temperature, hypoxia (a condition expected to intensify in CC scenarios) synergistically increased NP toxicity in *D. magna* through oxidative stress-mediated pathways and impaired energy metabolism (Lee et al., 2024), suggesting that deoxygenation events accompanying stratification may dramatically worsen NP impacts.

In aquatic macrophytes, the temperature-NP toxicity relationship parallels that observed in microinvertebrates. Bottega et al. (2024) reported that elevated temperature enhanced NP uptake and exacerbated histological and photosynthetic impairments in *Azolla filiculoides*, likely through increased cellular permeability and metabolic stress. These findings support an emerging pattern across trophic levels wherein warming amplifies NP bioavailability and toxicity.

Fish studies revealed more complex temperature-dependent outcomes than other organism groups, suggesting species-specific or life-stage-specific adaptations to warming. In most instances, rising temperatures worsened NP toxic effects including DNA damage, hepatic necrosis, and metabolic disruption in both model (*Danio rerio*; Şenol et al., 2023; Sulukan et al., 2022) and ecologically relevant species (*Oncorhynchus niloticus*; Soliman et al., 2025). However, some evidence suggests that adaptive physiological responses may partially alleviate NP toxicity under warming: Trevisan et al. (2025) found that elevated temperature enhanced myocardial contractility in zebrafish despite increases in oxidative stress, indicating that compensatory mechanisms can partially alleviate NP-induced cardiovascular toxicity. Beyond temperature, salinity (another CC-related variable subject to shifts in freshwater systems due to altered precipitation and evaporation patterns) was identified as a NP toxicity modulator in *O. niloticus* (Iqbal et al., 2025), with elevated salinity exacerbating NP-induced cellular degeneration and immune activation.

The severity of data scarcity regarding CC interactions with NP cannot be overstated. Notably, no studies were identified addressing CC-derived stressors in freshwater mollusks or macrocrustaceans; two groups of considerable ecological and economic importance.

Collectively, emerging evidence demonstrates that rising temperatures represent a consistent and significant amplifier of NP toxicity across freshwater organisms. The magnitude of this amplification (exemplified by the four-fold shift in LC_50_ with a modest 5 °C increase) underscores the urgent need to integrate climate scenarios into ecotoxicological risk assessment frameworks.

### Trojan Horse-induced mechanism by NP under co-exposure with other stressors

The Trojan Horse effect constitutes one of the most relevant toxicity mechanisms associated with NP. This phenomenon occurs when NP adsorb co-existing contaminants onto their surface and subsequently facilitate their translocation across biological barriers, thereby enhancing bioavailability and tissue accumulation beyond what would occur with the pollutant alone (Zhang and Xu, 2022). Several physicochemical properties of NP favor this mechanism, including their high surface area-to-volume ratio, hydrophobic character, and colloidal behavior. Once internalized, NP can release adsorbed contaminants within tissues, leading to localized toxicity that would not occur through waterborne exposure alone.

Our review identified Trojan Horse-mediated effects across multiple taxonomic groups and contaminant classes. In microcrustaceans, Monikh et al. (2020) demonstrated that NP enhanced silver uptake *in Daphnia magna*, with smaller particles (300 nm) showing greater carrier capacity than larger ones (600 nm). Similarly, Wang et al. (2024) reported increased carbamazepine bioaccumulation in triple mixtures containing NP and fulvic acids.

In fish, this mechanism has been documented for diverse contaminants: polycyclic aromatic hydrocarbons were transported to specific organs via NP in zebrafish (Trevisan et al., 2020; Zhang and Goss, 2021), methylmercury was redirected toward the head and eyes causing enhanced neurotoxicity (Oger et al., 2025), and antibiotics showed increased tissue penetration when co-administered with NP (Barreto et al., 2023a; Kamel et al., 2025). In macrocrustaceans, NP facilitated heavy metal bioaccumulation in *Eriocheir sinensis*, potentially enabling contaminant transfer through food webs (Banaee et al., 2025; Xu et al., 2025).

Importantly, dissolved organic matter (DOM) can modulate the Trojan Horse effect by competing for NP surface binding sites. Several studies demonstrated that humic and fulvic acids reduced contaminant sorption onto NP and mitigated toxicity in *D. magna* (Fadare et al., 2020; Lin et al., 2020; Monikh et al., 2020). However, DOM can also enhance bioavailability under certain conditions, as shown for carbamazepine in triple-mixture experiments (Wang et al., 2024), indicating that outcomes depend on complex interactions among NP size, DOM concentration, and contaminant properties.

### Polycyclic aromatic hydrocarbons, humic acids, and NP

The interaction between NP, PAH, and humic acids (HA) provides key mechanistic insights into contaminant modulation by NP. Due to their hydrophobic surface, NP readily sorb PAH from the surrounding medium, effectively acting as concentration vectors that deliver organic pollutants to biological membranes upon internalization (Trevisan et al., 2020; Zhang and Goss, 2021). Smaller NP exhibit greater sorption capacity per unit mass due to their higher surface area-to-volume ratio, explaining the size-dependent enhancement of PAH uptake and EROD induction observed in fish tissues (Zhang and Goss, 2021). Conversely, HA compete with PAH for NP surface binding sites and can form eco-coronas that alter particle surface chemistry, reduce hydrophobic interactions, and consequently decrease PAH bioavailability (Fadare et al., 2020; Lin et al., 2020; Zhang and Goss, 2021). This competitive sorption mechanism explains the mitigative effect of natural organic matter consistently reported across taxa, from microcrustaceans (Lin et al., 2020) to fish (Zhang and Goss, 2021).

## Gaps of knowledge and concluding remarks

Plastic-waste pollution constitutes a growing threat to freshwater ecosystems, where nanoplastics (NP) co-occur with other environmental pollutants and are subject to climate change (CC)-derived stressors. Despite growing evidence of NP toxicity, the combined effects of these multiple stressors on freshwater organisms have been insufficiently addressed compared to marine environments.

Based on the cases of study analyzed, this review reveals that freshwater algae, microinvertebrates, and fish are the most studied groups, yet taxonomic diversity remains limited, with *Chlorella* spp., *Daphnia magna*, and *Danio rerio* dominating the literature. Metals and pesticides were the most frequently studied co-stressors, whereas emerging pollutants such as pharmaceuticals and nanomaterials remain underexplored. Interaction outcomes were highly context-dependent, ranging from synergistic to antagonistic, depending on the species, co-stressor type, NP characteristics, and environmental conditions. Notably, synergistic interactions were the most frequently reported, particularly under co-exposure to metals.

The Trojan Horse mechanism emerged as a central toxicity mechanism, whereby NP adsorb co-existing contaminants and facilitate their translocation across biological barriers, enhancing bioavailability and tissue-specific accumulation. This mechanism was documented across multiple taxa and contaminant classes, from PAH transport to fish organs to enhanced metal bioaccumulation in macrocrustaceans. Conversely, dissolved organic matter, particularly humic acids, modulated NP toxicity through competitive sorption and eco-corona formation, reducing contaminant bioavailability. Oxidative stress, disruption of energy metabolism, immune suppression, and gut microbiota dysbiosis were consistently identified as key toxicological pathways across organism groups.

Significant knowledge gaps remain. CC-derived stressors are not represented within mollusk and macrocrustacean groups; notably, only two reports were found for mollusks assessing metal co-exposure to NP. While algae are well represented, CC-derived stressors have been poorly addressed for this group. The low taxonomic diversity of model species limits ecological extrapolation, particularly for environmentally relevant species representative of natural freshwater communities. Future research should prioritize multi-stressor experimental designs that incorporate environmentally realistic concentrations and CC scenarios. Furthermore, the development of predictive tools, including machine learning-based approaches, could help anticipate NP interaction outcomes under varying environmental conditions, thereby supporting more robust ecological risk assessment frameworks for freshwater ecosystems in a changing world.
