# Supplementary material for: Triple threat: a review on nanoplastic ecotoxicity, pollutant co-exposures, and climate change in freshwater organisms
Source: Front Physiol. 2026 Apr 16;17:1808330. doi: 10.3389/fphys.2026.1808330 (PMC13128419; doi:10.3389/fphys.2026.1808330)
Supplement: Supplementary file 1 [file DataSheet1.pdf]

SUPPLEMENTARY MATERIAL

Triple threat: A review on nanoplastic ecotoxicity, pollutant co-exposures, and climate change in freshwater organisms

Analía Ale[1*], Victoria S. Andrade[2], Lidwina Bertrand[3,4], María Florencia Gutierrez[2,5]

[1] Cátedra de Toxicología, Farmacología y Bioquímica Legal, Facultad de Bioquímica y Ciencias Biológicas, Universidad Nacional del Litoral (FBCB-UNL), CONICET, Santa Fe, Argentina.

[2] Instituto Nacional de Limnología (CONICET-UNL). Santa Fe, Argentina.

[3] Laboratorio de Investigaciones en Contaminación Acuática y Ecotoxicología (LICAE), Centro de Investigaciones Bioquímicas e Inmunología (CIBICI), CONICET, Córdoba, Argentina.

[4] Facultad de Ciencias Químicas, Departamento de Bioquímica Clínica, Universidad Nacional de Córdoba (UNC), Córdoba, Argentina.

[5] Escuela Superior de Sanidad "Dr. Ramón Carrillo" (FBCB-UNL), Ciudad Universitaria, Santa Fe, Argentina

[*] Correspondence: aale@fbcb.unl.edu.ar

Table S1. Summary of the cases of study analyzed per freshwater organisms' group: algae, plant, microinvertebrate, mollusk, macrocrustacean, and fish exposed to nanoplastics (NP) under co-exposure with other stressors.

Species	Life stage	Exposure time	NP size and concentration	Size checked?	Co-exposure conditions	Endpoints analyzed	Main results	Reference
ALGAE								
Euglena gracilis	Not applicable	4 days	100 nm<br>50 µg/L	Yes	Cd<br>50 µg/L	Growth inhibition, oxidative stress	Synergistic effects were evidenced under co-exposure in terms of microalgal growth by 28.76%. Superoxide dismutase (SOD), peroxidase (POD) and extracellular polymeric substances were distinctly enhanced in co-exposure treatments, indicating that cellular antioxidant defense responses were activated. Significant disruption to carbohydrate and purine metabolism-related pathways.	Cao et al. (2022)
Chlorella sp. TJ6-5	Not applicable	6 days	500 nm<br>47.7 to 99 mg/L	Yes	Cu<br>88.5 µM	Oxidative stress, morphological changes	NP increased the toxicity of Cu and induced oxidative stress and caused morphological and ultrastructural changes in microalgae.	Wan et al. (2021)
Pseudokirchneriella subcapitata NIES-35	Not applicable	6 days	500 nm<br>47.7 to 99 mg/L	Yes	Cu<br>92.4 µM	Oxidative stress, morphological changes	NP increased the toxicity of Cu and induced oxidative stress and caused morphological and ultrastructural changes in microalgae.	Wan et al. (2021)
Chlamydomonas reinhardtii	Not applicable	3 days	93 nm<br>200 mg/L	Yes	Clothianidin (pesticide)<br>35.2 to 211,4 mg/L	Growth inhibition tests	The mixture caused antagonistic interactions.	Bandeira et al. (2025)
Microcystis aeruginosa	Not applicable	3 days	93 nm<br>200 mg/L	Yes	Clothianidin (pesticide)<br>46.9 to 281 mg/L	Growth inhibition tests	The mixture caused antagonism to additivity.	Bandeira et al., (2025)
Scenedesmus obliquus	Not applicable	3 days	200 nm<br>25 mg/mL	No	Extra polymeric substances (EPS) (natural exudate, total organic carbon content: 13.185 mg/L)	Morphology, oxidative stress	Aged NP had significantly lesser toxic effects compared to pristine ones. A substantial decline in the levels of the reactive oxygen species, activity of the oxidative stress markers (SOD and catalase -CAT-) and cell membrane damage corroborated well with observed increase in the cell viability. Improved photosynthetic efficiency parameters correlated well with decreased levels of the reactive species in the cells.	Giri and Mukherjee (2021)
Chlorella vulgaris	Not applicable	3 days	200 nm<br>25 to 400 mg/L	Yes	Humic acids (natural organic matter, NOM)	Biomass and chlorophyll a	Humic acids significantly mitigated NP toxicity to algae biomass and chlorophyll content. Morphological changes included reduction of algal size and formation of aggregates.	Hanachi et al., (2022)

Species	Life stage	Exposure time	NP size and concentration	Size checked?	Co-exposure conditions	Endpoints analyzed	Main results	Reference
Chlorella pyrenoidosa	Not applicable	4 days	600 nm<br>1 mg/L	Yes	Ibuprofen (IBU, pharmaceutical)<br>5 to 100 mg/L	Bioaccumulation, biodegradation and enantioselectivity, and growth rate, chlorophyll a, total antioxidant capacity (TOC), reactive oxygen species concentration (ROS) and lipid peroxidation (malondialdehyde content, MDA)	The inhibitory effect of IBU on algae growth was alleviated in the presence of NP. NP caused decreased oxidative stress induced by IBU. Treatments with NP led to a decreased bioaccumulation and accelerated biodegradation of IBU and enhanced removal in the medium	Wang et al. (2020)
Scenedesmus obliquus	Not applicable	3 days	100 nm<br>10, 30, 50, 70, and 100 mg/L	Yes	CO_2, temperature, light (climate change variables)	Growth inhibition	Concentration-dependent inhibition of algal growth in the presence of NP, highlighting a threat to primary productivity in aquatic ecosystems. Crucial interactions between NP and climate change-derived stressors: low temperature and ambient CO_2 exacerbated damage induced by NP, while elevated CO_2 and warmer temperatures reflecting attenuated NP toxicity.	Yang et al. (2020)
Pseudanabaena biceps	Not applicable	4 days	800 nm<br>1.1 to 702.6 mg/L	No	Polybrominated diphenyl ethers (PBDE) (brominated flame retardants)<br>12.5 to 800 mg/L	Growth inhibition, oxidative stress, protein content	Co-exposure had more hazardous effects on cyanobacteria than changing climate conditions. Joint exposure resulted in lower toxicity. The mixture had antagonistic effects, attributing to the aggregation of NP, the adsorption of PBBDE, and the wrapping of both contaminants by released extracellular polymeric substances.	Xin et al. (2022)
Chlorella vulgaris	Not applicable	3 days	100 nm<br>1 mg/L	Yes	Cd<br>0.4 mg/L	Growth inhibition	NP were excluded outside the algal cells. The role of NP as carriers in Cd toxicity was negligible. NP increased the toxicity of Cd by altering cell wall polysaccharide contents. High dose Cd facilitated NP entering algal cells by loosening cell walls.	Zhang et al. (2024)
Scenedesmus obliquus	Not applicable	3 days	100 to 200 nm<br>2 mg/L	Yes	TiO_2NP (titanium dioxide nanoparticles)<br>0.025, 0.25, and 2.5 mg/L	Growth inhibition, oxidative stress, antioxidant activity, morphology	TiO_2NP and fluorescent NP decrease the algae viability and induced oxidative stress. Decrease in photosynthetic yield and esterase activity was observed. TiO_2NP and NP altered algal	Das et al. (2022)

Species	Life stage	Exposure time	NP size and concentration	Size checked?	Co-exposure conditions	Endpoints analyzed	Main results	Reference
							morphology.	
Chlorella vulgaris	Not applicable	-			Biochar<br>Pb (concentration not available)	Cell growth, oxidative stress, chlorophyll	Biochar has a mitigating effect on the algal toxicity of NP	Li et al. (2024)
Chlorella vulgaris	Not applicable	4 days	90 nm<br>0.125, 0.25, 0.5, and 1 mg/L	yes	Pb<br>20 and 80 μg/L	Size reduction and cellular aggregation	Overall, synergistic effects were evidenced under co-exposure.	Khoshnamvand et al. (2024)
Chlorella vulgaris	Not applicable	21 days	0.05 to 0.4 mg/L	Yes	Atrazine (ATZ, pesticide)<br>10 μg/L	Biomass, chlorophylls a and b, total antioxidant, total protein, oxidative stress (SOD, MDA)	The synergistic toxicity of the NP+ATZ group could be due to their enhanced bioavailability for algal cells.	Khoshnamvand et al. (2024)
Scenedesmus obliquus	Not applicable	3 days	400 nm<br>1 mg/L	Yes	Tetrabromobisphenol A (TBBPA) (flame retardant)<br>1 mg/L	Growth inhibition, oxidative stress, and antioxidant activity	The mixture of NP+TBBPA showed enhanced growth inhibition, oxidative stress, and antioxidant activity while reducing photosynthetic pigment levels compared to pristine TBBPA.	Dayana et al. (2025)
Scenedesmus obliquus	Not applicable	15 days	100-200 nm<br>0.01 to 1 mg/L	Yes	Diclofenac (pharmaceutical)<br>1 mg/L	Oxidative stress, antioxidant enzyme activity, and photosynthetic pigment content	Antagonistic effects for low-dose combinations of DCF and a synergistic effect for high-dose combinations.	Christudoss et al. (2024)
Microcystis aeruginosa	Not applicable	3 days	100-475 nm<br>5 to 100 mg/L	yes	Hydrogen peroxide<br>3297 mg/L, 30% w/w	Inhibitory effect	NP weakened the inhibitory effects of H_2O_2 on cell abundance and microcystin production	Guo et al. (2021)
Microcystis aeruginosa	Not applicable	4 days	80 nm<br>0.5 to 5 mg/L	Yes	Cd<br>0.2 to 2 mg/L	Chlorophyll content, and enzymatic activities	Synergistic effect: NP+Cd stimulated the production of microcystin-LR.	Wang et al. (2023)
Microcystis aeruginosa	Not applicable	3 days	40 nm<br>50 mg/L	Yes	$CuSO_4$ (algaecide)<br>0.05 to 5 mg/L	Inhibition, microcystin production (MC-LR)	Co-exposure of NP and CuSO4 exacerbates the negative effects of the salt on photosynthetic activity and altered algae metabolism. Most importantly, co-existence of NP with $CuSO_4$ decreased MC-LR content in the water.	Mamatimin et al. (2025)
Scenedesmus obliquus	Not applicable	4 days	50 to 100 nm	No	Sulfapyridine (SPY), sulfamethazine (SMR), sulfamethoxypyridazine (SMP), and sulfamethoxazole (SMZ) (antibiotics)	Physiological and biochemical parameters	Toxicity of the binary mixtures was generally lower than that of the individual exposures, suggesting that the presence of NP reduced the toxicity of the antibiotics.	Yang et al. (2025)

Species	Life stage	Exposure time	NP size and concentration	Size checked?	Co-exposure conditions	Endpoints analyzed	Main results	Reference
Chlamydomonas reinhardtii	Not applicable	3 days	500 nm<br>100 mg/L	Yes	Sulfamethoxazole (SMX, antibiotic)<br>2.5 to 10 mg/L	Growth inhibition	Co-exposure of NP+SMX exhibited a strong antagonistic/mitigative effect under light. NP could adsorb more SMX under light, thereby alleviating SMX toxicity to the algae	Wang et al. (2023)
Chlorella pyrenoidosa	Not applicable	3 days			AgNP (silver nanoparticles)<br>(concentrations not available)	Growth inhibition, oxidative stress	Antagonistic effect of NP and AgNP: toxicity of AgNP to the algae was reduced likely due to adsorption by NP. The adsorption between AgNP and PSNPs led to increased particle size, reducing the bioavailability of nanoparticles and thus the toxicity of binary mixture on the algae.	Li et al. (2025)
PLANTS								
Hydrilla verticillata	Not applicable	16 days	100 nm<br>10 mg/L	Yes	Bisphenol F (BPF)<br>1 and 10 mg/L	Growth inhibition, chlorophyll content, oxidative stress	Co-exposure of BPF+NP decreased the growth rate and chlorophyll content. They trigger antioxidant responses such as increased activities of SOD, POD, and glutathione S-transferase (GST), and altered MDA levels and decreased CAT activity. The combination of NP+ BPF produced an antagonistic response for plant growth. BPF presence increased the adsorption of NP in the leaves surface.	Yu et al. (2022)
Azolla filiculoides	Not applicable	7 days	30 nm<br>50 mg/L	No	Temperature<br>25 and 35 °C	Uptake, development, physiology, histology	Histological, morphological, and photosynthetic parameters worsened under co-exposure, in accordance with the increased uptake of NP under higher temperature	Bottega et al. (2024)
Lemna minor	Not applicable	7 days	93 nm<br>200 mg/L	Yes	Clothianidin (pesticide)<br>1 to 1000 mg/L	Growth inhibition	The mixture caused antagonism to additivity	Bandeira et al. (2025)
MICROINVERTEBRATES								
Daphnia magna	Neonate	2 and 21 days	92.6 nm<br>Mortality: 75 mg/L<br>Growth and reproduction: 4.97 mg/L<br>Behavior: 50	Yes	Clothianidin (pesticide) mortality: 67 mg/L growth: 17.83 mg/L	Mortality, multigenerational growth and reproduction, swimming behavior	NP caused mortality and alterations in growth reproduction and swimming behavior the insecticide caused mortality and inhibited growth. Antagonism was predominant under mixture conditions.	Bandeira et al. (2025)

Species	Life stage	Exposure time	NP size and concentration	Size checked?	Co-exposure conditions	Endpoints analyzed	Main results	Reference
			mg/L					
	Neonate	2 days	44.1 nm<br>0.001-100 mg/L	Yes	Diphenhydramine (DPH, pharmaceutical)<br>DPH LC50: 4 mg/L<br>Mixture 1 LC50: 0.001 mg/L DPH + 100 mg/NP<br>Mixture 2 LC50: 3 mg/L DPH + 0.001 mg/L NP	Mortality, oxidative stress, acetylcholinesterase activity (neurotoxicity)	Both mixtures increased lipid peroxidation levels. Synergistic interactions occurred at environmentally relevant concentrations. NP+DPH induced oxidative damage, whereas no effect was observed at single exposures. With the increase of NP concentration, the DPH concentration causing 50% of effect (EC50) for organisms' immobilization decreased to 0.001 mg/L.	Barreto et al. (2023)
	Neonate	Not reported (until second generation reproduction)	154.1 nm<br>5 µg/L	Yes	Temperature: 20 and 24°C, plus three daily temperature fluctuations (DTF, constant: 0 ºC, low 5 ºC, high 10 ºC)	Variables were analyzed in the second generation: reproduction,<br>critical thermal maximum, locomotor capacity loss, activity of cytochrome P450,<br>energy budget	Effects of NP were evidenced only at warming and/or DTF conditions. NP increased fecundity, heat tolerance, energy storage, net energy budget, and cytochrome P450 activity, and decreased the energy consumption when tested under an increased NP or DTF. Hormesis effect induced by NP under warming conditions was suggested.	Chang et al. (2023)
	Neonate	2 and 4 days	100 nm<br>1-400 mg/L	Yes	Organic matter (humic acids, HA)<br>1, 5, 10, 20 and 50 mg/L	Mortality, gene expression of detoxification-related genes (CAT, HSP70, p-gp)	NP lethal effects dramatically decreased by the presence of HA. NP upregulated all the examined genes while HA diminished the change appreciably. HA adsorption on the NP was evidenced and formed a corona without causing agglomeration or precipitation; however, it changed the particle distribution in the organisms thus leading to alleviated toxicity.	Fadare et al. (2020)
	Neonate	0.5, 1, and 21 days	277 nm<br>0.5, 1, 21 mg/L	Yes	Pyriproxyfen (pesticide)<br>EC50-24 h: 0.24 mg/L<br>Chronic exposure: 100 and 200 ng/L	Mortality, growth, reproduction, and uptake	NP addition within 24 h reduced the acute toxicity of pyriproxyfen. The pesticide significantly disturbed the growth and reproduction of the organisms after chronic exposure. Alleviated effects were assumed under co-exposure of both NP and the pesticide, together with a lower particle uptake.	Jia et al. (2024)
	Adult	2 and 21 days	50 nm<br>LC50 alone:	No	Hypoxia<br>Result of LC50: 1,2 mg/L O_2<br>Chronic assay: 2 mg/L O_2	Mortality, oxidative stress, growth, and reproduction	Co-exposure of hypoxia and NP exhibited a negative synergy that increased reactive oxygen species concentration and the related	Lee et al. (2024)

Species	Life stage	Exposure time	NP size and concentration	Size checked?	Co-exposure conditions	Endpoints analyzed	Main results	Reference
			44.92 mg/L LC50 with hypoxia: 19.5 mg/L Chronic assay: 5 mg/L				antioxidant enzyme activities. The effects lead to more severe reproductive and growth impairments in the organisms compared to single-stressor exposure.	
	Adult	1.5 days	117 nm 1 mg/L	Yes	Humic acid (HA, 100 mg/L) Polycyclic aromatic hydrocarbons (PAH: acenaphthene, fluorene, phenanthrene, anthracene, fluoranthene and pyrene) (concentrations not available)	PAH bioaccumulation by modeling calculation and experimentation	NP matrix would retard the intestinal uptake process, especially for the less hydrophobic PAH; while the HA or the HA-NP matrix would facilitate the mass transfer of PAH from the matrix to lipids in the organisms' gut.	Lin et al. (2020)
	Neonate	5 days	420 nm Concentration stated as 1×10^3, 1×10^6, and 1×10^9 particles/L	Yes	Enrofloxacin (antibiotic) 10 and 100 ng/L	Reproduction, growth, gut microbiota	NP or the antibiotic alone negatively affected most of the measured parameters (body volume, clutch size, egg volume, and respiration rate of the gut microbiota), and there was a significant negative interaction in the effect of both stressors on all of the measured parameters.	Maszczyk et al. (2022)
	Adult	3 days	300 and 600 nm Concentration stated as 6.74×10^{10} particles/L	Yes	$AgNO_3$: 0, 1, 2, 5 and 10 µg/L Dissolved organic matter (DOM): 0, 1, 10 and 50 mg/L	Mortality, accumulation, oxidative stress	DOM presence increased the sorption of Ag^+ onto 300 nm-sized NP-based debris (NP-D) but decreased the sorption onto 600 nm-sized NP-D. Mixture of NP-D and Ag^+ (1 mg/L) induced toxicity for both NP sizes. DOM inhibited the combined toxicity of Ag^+ and NP-D regardless of the size and chemical composition. NP-D increased the uptake of Ag^+ in the organisms. DOM decreases the bioconcentration factor of Ag^+ in all cases. NP-D enhanced oxidative stress of Ag^+ and the related enzyme activities to eliminate the ROS. NP-D size of NPD highly influences the Trojan horse mechanism (particularly in the case of 300 nm-sized NP). DOM inhibited oxidative stress.	Monikh et al. (2020)
	Neonate	2 and 21 days	73 nm EC50-48 h:	Yes	Glyphosate (Gly, pesticide) EC50-48 h: 89.33 mg/L	Mortality, swimming behavior, reactive oxygen	Joint toxicity resulted increased under co-exposure, thus indicating a synergy.	Nogueira et al. (2022)

Species	Life stage	Exposure time	NP size and concentration	Size checked?	Co-exposure conditions	Endpoints analyzed	Main results	Reference
			244 mg/L			species concentration (ROS), longevity, reproduction, multigenerational effects	The mixture increased the immobility and ROS production, and decreased swimming activity. Multigenerational responses indicated that the exposure of F0 daphnids exposed to the mixture induced effects in the F1 and F2 reproduction parameters in the recovery tests.	
	Neonate	2 days	100 nm<br>10, 50, 100, 200, and 400 mg/L	No	Triclosan (personal care product)<br>10, 50, 100, 200, and 400 µg/L	Mortality	Co-exposure to triclosan and NPs increased the mortality rate, and the combination of caffeine and NP did not show a clear pattern of interaction.	Pashaei et al. (2023)
	Neonate	2 days	100 nm<br>10, 50, 100, 200, and 400 mg/L	No	Caffeine<br>10, 50, 100, 200, and 400 µg/L	Mortality	Co-exposure to triclosan and NP increased the mortality rate, and the combination of caffeine and NP did not show a clear pattern of interaction.	Pashaei et al. (2023)
	Neonate and adult	1, 2, and 21 days	50 nm<br>LC50-48 h at 23°C: 4.413 mg/L<br>at 28°C: 1.062 mg/L<br>Chronic bioassay: 1, and 2 mg/L	No	Temperature<br>23 and 28°C	Mortality, accumulation, growth, reproduction, oxidative stress, gene expression (associated with oxidative stress, protein expression related to apoptosis and cell proliferation)	Increased temperatures augmented NP driven lethality. Elevated temperature induced the elimination of NP in the organisms, resulting in a reduction of accumulation at 28 °C. Co-exposure to both higher temperatures and NP resulted in a drastic decrease in reproductive performance. Oxidative stress increased in a temperature-dependent manner; it was stimulated by both stressors, leading to increased levels of reactive oxygen species and antioxidant enzyme activity supported by upregulation of antioxidant enzyme-related genes under combined NP exposure and elevated temperature. Activation of the p38 mitogen-activated protein kinase signaling pathway was induced by exposure to NP at high temperatures, which supported the decline of the reproductive capacity.	Sanpradit et al. (2024)
	Neonate	2 days	30, 200, 1000 nm<br>EC50-48 h for 30 nm-sized NP: 1-65 mg/L<br>for 200 nm-	No	Halogenated polycyclic aromatic hydrocarbons (HPAH)<br>EC50-48 h: 0.12–0.22 mg/L	Mortality	NP with sizes beyond *D. magna* feeding range had little toxicity: toxicity decreased with increasing particle size. HPAH alone were highly toxic. The mode of action of PS and HPAHs was antagonistic. The size-effect of the NP operated via two mechanisms: the	Sun et al. (2024)

Species	Life stage	Exposure time	NP size and concentration	Size checked?	Co-exposure conditions	Endpoints analyzed	Main results	Reference
			sized NP: 17.8 mg/L<br>for 1000 nm-sized NP: 189 mg/L				inherent toxicity and the sorption of pollutants by NP; the former impacts the combined toxicity more than the latter. In the binary mixed system, the larger the particle size and the higher the proportion of NP in the system, the less toxic the system was.	
	Adult	2 days	50, 100, and 1000 nm<br>Alone: 5 mg/L<br>Co-exposure: 2.5 mg/L	Yes	Fulvic acid (FA)<br>5.0 mg/L<br>Carbamazepine (CBZ, anticonvulsant pharmaceutical)<br>100 µg/L	Bioavailability and accumulation	Both NP and FA reduced the elimination rate of CBZ. The availability of CBZ was determined by the hydrodynamic particle size of NP, whereas the bioavailability to *D. magna* depended on the intrinsic particle size. CBZ bioavailability was greater in co-exposed matrices due to the attenuated sorption of NP to CBZ by FA modification. Co-exposure of NP and FA resulted in a higher bioaccumulation factor of CBZ, probably due to the desorption and reabsorption of particle-associated CBZ.	Wang et al. (2024)
	Adult	2 days	140 nm<br>5 µg/L	Yes	Temperature<br>20 and 24°C<br>nd three daily temperature fluctuations (DTF: constant 0 ºC, low 5 ºC high 10 ºC)	Heartbeat rate, thoracic limb activity, swimming speed, feeding rate, activity levels of Cytochrome P450 (CytP450), acetylcholinesterase (AChE), mitochondrial electron transport system (ETS), and hemoglobin concentration (Hb)	Overall, NP exposure reduced heartbeat rate, thoracic limb activity and feeding rate, and increased CytP450, ETS activity and Hgb concentrations. Higher mean temperature (MT) and DTF enhanced the effects. Clones originating from their respective sites performed better under their native temperature conditions, indicating local thermal adaptation. Warm-adapted *D. magna* showed stronger NP-induced increases in CytP450, ETS activity and Hb concentrations under MT 24 ºC, while cold-adapted *D. magna* showed stronger NP-induced decreases in heartbeat rate, thoracic limb activity and feeding rate under high temperature.	Xu et al. (2024)
	Neonate	1 day	240 nm<br>50, 500, and 5000 µg/L	Yes	Chlorpyrifos (CPF, pesticide)<br>0, 0.08, 0.8, 8, 40.7, 162.8, 325.7, 651.4, and 1032.8 µg/L	mortality, uptake and accumulation, Heart rates	NP alone had no acute toxicity, while CPF exhibited high toxicity, with an LC50 of 50.8 µg/L. However, in the presence of 50 µg/L NP, the LC50 increased to approximately 400 µg/L, indicating an antagonistic effect. The adsorption of CPF onto NP can reduce the	Zhang et al. (2024)

Species	Life stage	Exposure time	NP size and concentration	Size checked?	Co-exposure conditions	Endpoints analyzed	Main results	Reference
							concentration of free CPF, leading to aggregation and sedimentation of CPF-PS-NP clusters, thereby decreasing the uptake of the pesticide.	
Hydra viridissima	Adult	4 days	Polyhydroxybutyrate (PHB) 200 nm	Yes	Cd 0.01-1000 µg/L	Mortality, morphological changes, and feeding behavior	NP were capable of modulating the metal toxicity: co-exposure produced a lower toxicity effect than the metal alone.	Santos et al. (2024)
	Adult	4 days	Polyhydroxybutyrate (PHB) 200 nm	Yes	Zn 0.01-1000 µg/L	Mortality, morphological changes, and feeding behavior	NP were capable of modulating the metal toxicity: response pattern of the combination was similar to that of the metal alone (i.e. no interactive effect).	Santos et al. (2024)
	Adult	4 days	Polyhydroxybutyrate (PHB) 200 nm	Yes	Cu 0.01-1000 µg/L	Mortality, morphological changes, and feeding behavior	NP were capable of modulating the metal toxicity: interaction produced higher effect than the metal alone.	Santos et al. (2024)
Brachionus calicyflorus	Adult	Not reported	Amidine and carboxyl-stabilized poly styrene NP 200 nm	Yes	Organic matter (OM)	Mortality	Both amidine and carboxyl NP were ingested and concentrated mainly in the stomach. Amidine NP was more toxic than carboxyl one. Organic matter reduced significantly the toxicity of both NP. The eco-corona modulated the surface charge and reduced toxicity to zooplankton.	Saavedra et al. (2019)
MOLLUSK								
Bellamya aeruginosa	Adult	28 days	82.48 nm 100 mg/kg	Yes	Cu 5 and 25 mg/kg	Bioaccumulation, DNA damage, oxidative stress (malondialdehyde content -MDA-, superoxide dismutase enzyme activity), neurotoxicity (AChE enzyme activity), metallothionein (MT).	NP increased Cd bioavailability and facilitated Cd bioaccumulation. Cd contents in the NP+low-Cd and NP+high-Cd groups were significantly increased by 40% and 45% respectively, indicating that NP in the sediments could enhance Cd uptake by the snails. Under the sediment exposure, the role of NP as vectors to transfer Cd absorbed on NP to the snails after ingestion might be negligible. Co-exposure of NP+low-Cd or high-Cd produced greater DNA damage than the single Cd exposure. Co-exposure to NP+Cd enhanced oxidative and DNA damage and reduced MT levels. Co-exposure NP+high-Cd decreased MT levels and increased Cd	Luo et al. (2022)

Species	Life stage	Exposure time	NP size and concentration	Size checked?	Co-exposure conditions	Endpoints analyzed	Main results	Reference
							accumulation in the hepatopancreas. Overall, the IBRv2 index revealed that co-exposure increased toxic stress in the organisms compared to single exposure, thus indicating a synergistic effect.	
Corbicula fluminea	Adult	21 days (waterborne and dietary exposures)	205 nm<br>0.008-10 µg/L<br>Two kinds: polyesters (PET), polyvinylics (PS and PVC)<br>NP-L: nanoplastic with undefined shapes	Yes	$AlCl_3$<br>1 mg/L	Oxidative-stress-gene regulation, oxidative stress, neurotoxicity, filtration/ventilatory activity	Dietary exposure to Al ions triggered more gene modulations than waterborne exposure to NP-L. NP-PS were also more harmful than NP-L, but only at high concentrations. The effects of each treatment lasted until 7 days of depuration. Few effects were shown in terms of oxidative stress. Inhibition of acetylcholinesterase (AchE) was concomitant with an increase pf the filtration activity exposed to NP-L and NP-PS in case of dietary exposure, suggesting neurotoxic effects. By disturbing the ventilatory activity, NP could have direct effects on xenobiotic accumulation and excretion capacities.	Arini et al. (2023)
MACROCRUSTACEAN								
Macrobrachium rosenbergii	Not informed	14 days	100 nm<br>50-250 µg/L	Not informed	Heavy metal cocktail (HMC) (Fe, Zn, Cd, Ni, Pb, Mg, Mn, V, Co, Cu) 0.5 mg/L	Oxidative stress, metabolic profile	Co-exposure to NP+HMC induced oxidative stress, biochemical alterations, and bioaccumulation. This leads to increased toxicity, disrupted metal balance, oxidative stress, and impaired metabolism. Cellular damage and reduce shrimp survival. Increase in antioxidant enzyme activities and malondialdehyde levels, coupled with a decrease in total antioxidant capacity, highlighted significant oxidative damage in the shrimp hepatopancreas. Diminished energy reserves indicated a state of metabolic stress. Molecular-level disturbances, including protein oxidation, membrane damage, and nucleic acid degradation	Banaee et al. (2025)
Eriocheir sinensis	Juvenile	10 days	100 nm<br>Concentration stated as	No	Cd<br>15 µg/L	Morphological alterations, histology, gene expression (ALF1, ALF2, and Crus1),	NP+Cd group displayed intestinal wall edema. Exposure to NP and/or Cd induced oxidative stress, as evidenced by a significant increase	Che et al. (2024)

Species	Life stage	Exposure time	NP size and concentration	Size checked?	Co-exposure conditions	Endpoints analyzed	Main results	Reference
			1.0×10^{10} particles/L			oxidative stress and antioxidant capacity, intestinal microbiota, transcriptomic analysis	in lipid peroxide content, total antioxidant capacity, and peroxidase activity, and significant decreases in superoxide dismutase (SOD) and glutathione peroxidase (GSH-PX) activities. In addition, exposure to NP and/or Cd imbalanced the homeostasis of the intestinal microbiota. Transcriptomic and metabolomic analyses showed that ferroptosis, ABC transporters, phosphotransferase system, apoptosis, and leukocyte trans- endothelial migration were disturbed after exposure to NP and/or Cd. Co-exposure to NP and Cd might mitigate intestinal toxicity by decreasing oxidative stress and affecting these pathways.	
	Not informed	28 days	100-250 nm<br>100 µg/L	No	Pentadecafluorooctanoic acid (PFOA)<br>2, 20, and 200 µg/L	Histology, hepatopancreatic transcriptomics, non-target metabolomics, gut microbial analysis, gene expression, oxidative stress	NP+PFOA exposure had higher toxic effects compared to single PFOA exposure, manifested by greater oxidative stress and worse histological damage. NP mediates apoptotic and ferroptosis pathways exacerbating PFOA-induced hepatotoxicity. Co-exposure to NPs-PFOA exacerbated the detrimental effects of PFOA on the intestinal flora, e.g., lower diversity indices, and more differential species. Moreover, NPs may worsen the PFOA-induced intestinal inflammatory response by mediating the harmful bacterium *Fusobacterium ulcerans*.	Huang et al. (2025)
	Not informed	21 days	100 nm<br>Concentration stated as 1.0×10^{10} particles/L	Not informed	Phoxim (pesticide)<br>24 µg/L	Histology, oxidative stress (superoxide dismutase -SOD-, peroxidase -POD-), antioxidant capacity (T-AOC), gene expression, transcriptomic analysis,	NP and/or PHO caused intestinal toxicity. Exposure caused histopathological changes, such as an increase in intraepithelial lymphocytes. Furthermore, the decrease of SOD, POD, and T-AOC suggested that NP and/or PHO exposure induced intestinal oxidative stress. Pro-inflammatory gene expression and transcriptome analysis demonstrated that NP and/or PHO exposure led to the occurrence of intestinal inflammation. Additionally, the co-exposure	Che et al. (2024b)

Species	Life stage	Exposure time	NP size and concentration	Size checked?	Co-exposure conditions	Endpoints analyzed	Main results	Reference
							caused less tissue damage and mitigated the intestinal inflammation.	
	Adult	21 days	100 nm<br>Concentration stated as 1.0×10^{10} particles/L	Not informed	Phoxim (pesticide)<br>24 μg/L	Histology, intestinal microbiota, non-targeted metabolite profile, efflux cellular activity (ABC transporters)	In all the exposure groups there was vacuolar degeneration occurring in epithelial cells. The peritrophic membrane exhibited thinning after NP or PHO single exposure, while thickening was observed after co-exposure. Exposure to NP and/or PHO disrupted the intestinal microbiota homeostasis, as evidenced by the proliferation of pathogenic bacteria and suppression of beneficial bacteria. Exposure to NP and/or PHO led to alterations in the metabolic profile as well as several critical pathways. Among these, the upregulation of arachidonic acid metabolism, ABC transporters, and biosynthesis of amino acids was observed in both NP single exposure and co-exposure. NP and/or PHO exposure downregulated neuroactive ligand-receptor interaction. Reduction of some metabolites involved in the anti-inflammatory or antioxidant mechanism	Ding et al. (2025)
Eriocheir sinensis	Adult	21 days	100 nm<br>0.4 mg/L	Not informed	Cu<br>0.1 mg/L	Accumulation, histology, oxidative stress (enzyme activities: SOD, CAT, GSH-Px, GSH, lipid peroxidation: MDA levels), antioxidant capacity (T-AOC), immune indicators (ACP, AKP, LZM, MT, caspase-3 and caspase-9 activities), transcriptomic analysis,	Structural damage to gill tissues, elevated antioxidant parameters, and altered immune markers were observed. Compared with the single exposure to Cu, energy metabolism-related genes (TAT, TPI, HK) were down-regulated in the co-exposed group. GSH metabolism and cytochrome P450 were notably affected, and the combined exposure suppressed the expression of immune-related genes such as CYP450, GST, and UGT	Xu et al. (2025a)
Eriocheir sinensis	Adult	1, 7, 14 and 21 days	100 nm<br>0.4 mg/L	Yes	Cu<br>0.1 mg/L	NP accumulation and histology of hepatopancreas, oxidative stress (MDA, GSH, SOD, CAT, GST). immune response (AKP, LZM, caspase-3, caspase-8,	Time-dependent increased NP and Cu accumulation in the hepatopancreas. The concentration of Cu accumulation increased with longer exposure times in all the tested treatments. After 14 and 21 days, Cu accumulation in the hepatopancreas was	Xu et al. (2025b)

Species	Life stage	Exposure time	NP size and concentration	Size checked?	Co-exposure conditions	Endpoints analyzed	Main results	Reference
						caspase-9), MT induction, transcriptomic analysis	significantly greater in the Cu group than in the co-exposure treatment. NP and/or Cu induced serious histopathological changes in the hepatopancreas and led to an increase in antioxidant enzymes and a decrease in most immune-related enzymes. Transcriptome sequencing analysis revealed an affected energy supply explained by interfering with lipid metabolism and activation of antioxidant systems. Key genes in the immune system were activated to mitigate the hepatopancreatic toxicity by the combined exposure.	
	Not informed	21 days	100 nm Concentration stated as 1.0×10^{10} particles/L	Yes	Phoxim (PHO, pesticide) 24 µg/L	Histology, oxidative stress (enzyme activities of SOD, GST, POD), transcriptomic analysis.	Exposure to NP and/or PHO elevated the antioxidant enzyme activities, thus leading to downregulation of oxidative stress-related genes in the NP and PHO-exposure groups. Decrease in survival reached the lowest values in case of the co-exposed group. Mucus and mechanical barrier damage in the hepatic tubules were observed in single and co-exposure treatments. Mucin-related genes were downregulated. Altered expressions of oxidative stress-related genes, mucin-related genes, and TJ-related genes were also observed. Altered related genes may indicate disruption of innate immunity caused by co-exposure. Co-exposure displayed an exacerbating effect on hepatopancreas inflammation when compared to individual exposures.	Huang et al. (2024)
FISH								
Danio rerio	Embryo	1, 2 or 3 days	50 nm 0.1, 0.5, and 1 mg/L	No	Temperature 27 y 30 ºC	Cardiovascular toxicity by omics, particle accumulation and distribution	NP (0.1 mg/L) entered into the embryos caused cardiovascular toxicity at 27 ºC. This was explained by the downregulation of the	Duan et al. (2023)

Species	Life stage	Exposure time	NP size and concentration	Size checked?	Co-exposure conditions	Endpoints analyzed	Main results	Reference
							branched-chain amino acid and insulin signaling pathways owing to induced oxidative stress. Elevated exposure temperatures promoted the accumulation of NP in developing zebrafish, increased the levels of oxidative stress and enhanced the oxidative phosphorylation rate in mitochondria, thus resulting in an additive effect on the mortality of zebrafish larvae. Elevated exposure temperatures reduced the cardiovascular toxicity of NP. Multi-omics analyses revealed that elevated temperatures enhanced the myocardial contractility of larvae, thus reducing the cardiovascular toxicity of NP.	
	Embryo	4 days	0.1 µm<br>10 mg/L	Yes	AgNP (silver nanoparticles): 0, 0.5, 1, 2, 4, and 8 mg/L), Ag^{+}: 0, 10, 20, 40, 60, and 160 mg/L	Acute toxicity, oxidative stress, apoptosis, immunotoxicity, and metabolic capability	NP could act as a carrier of the co-existing AgNP. Lethality by AgNP was not significantly changed by the addition of NP. Alterations in gene expression related to antioxidant and metabolic capabilities caused by AgNP were significantly enhanced by the presence NP, which simultaneously lowered the apoptosis and immunotoxicity induced by AgNP. Therefore, the presence of NP suppressed the AgNP-induced genotoxicity, while the absorption and agglomeration of AgNP and the released Ag^{+} on NP may alleviate the toxicity.	Yan et al. (2023)
	Embryo	7 days	44 nm<br>0.1, 1, and 10 mg/L	No	Temperature<br>28 vs 32 ºC	Metabolism, development, oxygen consumption, ATP production, apoptosis, oxidative stress	32 ºC increased markers associated with metabolism and developmental timing (growth, hatching, heart rate, and feeding). Changes in apoptosis dynamics, oxygen consumption rates, and a decrease in mitochondrial content were detected as adaptive processes to temperature. 44 nm-sized NP alone did not alter development but decreased mitochondrial efficiency in ATP production and increased apoptosis in the heart. Exposure to 44 nm-NP and 32 ºC did not cause major	Trevisan et al., (2025)

Species	Life stage	Exposure time	NP size and concentration	Size checked?	Co-exposure conditions	Endpoints analyzed	Main results	Reference
							implications to survival, developmental success, or morphology; however, 44-nm NP mitigated the temperature driven change in heart rate, increased oxidative stress, and decreased the coupling efficiency of the less abundant and highly active mitochondria under heat stress.	
	Larvae	30 days	250 nm<br>1 mg/L	No	Methylmercury (MeHg)<br>1 and 10 µg/	MeHg accumulation, neurotoxicity (swimming behavior), proteomics (lipid metabolisms, oxidative stress, detoxification, myogenesis)	NP enhanced MeHg accumulation and redirected its distribution toward the fish's head and eyes. NP alone altered swimming activity, while MeHg induced mortality, reduced growth and diminished swimming activity. Proteomic analysis highlighted significant effects on lipid metabolism, oxidative stress, detoxification, myogenesis and catabolism. Although no light sensitivity deficits were detected through visual motor response testing, proteomic data suggested vision impairment in the mixture-exposed groups. High mortality rates were observed in the co-exposure, likely due to severe hypoactivity, which hindered feeding. This hypoactivity was linked to disrupted lipid metabolism, impaired neurotransmission, reduced ATP production, and neuroinflammation leading to neuronal degeneration. Overall, NP intensified MeHg neurotoxicity over prolonged exposure, significantly increasing mortality.	Oger et al. (2025)
	Adults	4 days	100 nm<br>25 mg/L	Yes	Temperature<br>28, 29, 30 °C	Neurotoxicity (behavior), metabolomics, NPs detection in brain	1 ºC-increase under co-exposure to NP affected the circadian rhythm in fish, caused damage to the brain, and significant changes in the intensity of 18 metabolites in different pathways. Overall, it was suggested that 1 degree increased the way for degeneration in the brain by disrupting some metabolic pathways, thereby significantly increasing the negative effects of NP on behavior.	Sulukan et al. (2022)
	Adults	4 days	100 nm	Yes	Temperature	Tissue damage and	Exposure to NP and temperature increased	Şenol et al.

Species	Life stage	Exposure time	NP size and concentration	Size checked?	Co-exposure conditions	Endpoints analyzed	Main results	Reference
			25 mg/L		28, 29 and 30 °C	metabolomics	DNA damage through stress-induced responses accompanied by degeneration, necrosis and hyperaemia in fish liver, and adhesion of lamellae, desquamation and inflammation in lamellar epithelium in gills. Metabolomic analyses also supported changes indicating protein and lipid oxidation, especially mediated by NP.	(2023)
Oncorhynchus niloticus	Adults	25 days	100 nm<br>2 µg/kg	No	Salinity<br>2.5 ppt, 5 ppt, 10 ppt, and 15 ppt)	Immunity, malondialdehyde levels (MDA), histopathology	NP exposure induced significant cellular degeneration and necrosis, with increasing salinity exacerbating immune activation and oxidative damage. Elevated cytokine levels indicated heightened inflammatory responses, while a marked increase in MDA suggested enhanced lipid peroxidation and oxidative stress. Histological assessments showed structural abnormalities in tests and liver, which were more pronounced at higher salinity levels. Overall, salinity modulates NP toxicity by influencing immune and oxidative stress responses, leading to aggravated tissue damage.	Iqbal et al. (2025)
	Adults	4 days	<100 nm<br>10 mg/L	Yes	Temperature<br>30, 32, and 34 °C	Enzyme tissue damage (aspartate aminotransferase -AST-, alanine aminotransferase -ALT-, alkaline phosphatase -ALP-), albumin, globulin, oxidative stress	NP and high temperature increased the levels of plasma total proteins, albumin, globulin, enzyme tissue damage markers (AST, ALT, ALP), creatinine, and uric acid. Oxidative stress markers in the liver, gills, and brain were increased in MDA concentration, and a decrease in glutathione reduced (GSH) concentration and catalase (CAT) activity in all groups. Synergistic cytotoxic effect of NP and temperatures on the metabolic and oxidative stress indices of fish were evidenced.	Soliman et al. (2025)
Oncorhynchus mykiss	Fingerlings	1 day	20 and 500 nm<br>200 µg/L<br>(200 nm: $1.848{\times}10^{10}$	Yes	Polycyclic aromatic hydrocarbon (PAH)<br>1 mg/L<br>Humic acids (referred as natural organic matter, NOM)	Bioaccumulation in tissue, EROD (ethoxyresorufin-O-deethylase) activity, mitigation by NOM	20 nm-sized NP increased PAH uptake and induced higher EROD activity in both the gill and liver when compared to larger NP with sorbed PHA, or with the PAH-exposed alone group. NOM reduced both PAH uptake and	Zhang and Goss (2021)

Species	Life stage	Exposure time	NP size and concentration	Size checked?	Co-exposure conditions	Endpoints analyzed	Main results	Reference
			particles/mL; 500 nm: 2.91×10^6 particles/mL)		0.1 mg/L		EROD activity, especially in the presence of 20 nm-sized NP, demonstrating the mitigating effects of NOM. Smaller NP can transverse the epithelia of fish gills and are found in the liver 24 h after initial exposure. Moreover, while phenanthrene can be depurated from the fish within 24 h in clean fresh water, the 20 nm particles remain in the liver and gill tissue.	
Clarias gariepinus	Juvenile	15 days	20 and 80 nm<br>5 mg/L for both sizes	Yes	Potassium clavulanate (CA, antibiotic)<br>100 ng/L	Apoptosis, hematology (hemoglobin levels -Hb-, hematocrit -Hct-, reb blood cells count -RBC-, white blood cell count -WBC-), DNA damage, histology in spleen	20 nm-sized NP alone increased erythrocyte apoptosis, while CA alone also caused significant DNA damage. Co-exposure to CA and NP, particularly the 20 nm-sized NP, produced the strongest erythrocyte apoptosis, DNA damage, and declines in Hb, Hct, RBC, and WBC. These hematological and genotoxic effects persisted after recovery, indicating incomplete or delayed reversal of toxicity. Spleen histopathology showed vascular congestion, hemorrhage, fibrosis, and lymphoid depletion, especially in co-exposed fish. The results demonstrate size-dependent synergistic interaction, suggesting that smaller NP amplify CA-induced cytotoxicity and genotoxicity through a Trojan horse mechanism.	Kamel et al. (2025)

Table S2. Summary of geographic distribution of the included studies. Countries were determined based on the institutional affiliation of the corresponding author as listed in each publication. When studies involved international collaboration, multiple countries were considered.

Country	Number of study entries
China	32
Canada	9
Brazil	5
India	4
Belgium	3
Malaysia	2
Iran	2
Italy	2
Portugal	2
South Korea	2
France	2
Turkey	2
Egypt	2
Poland	1
Netherlands	1
Lithuania/Latvia	1
Thailand	1
Switzerland	1
USA	1
Indonesia	1

Figure S1. Flow diagram of the literature search and study selection process.

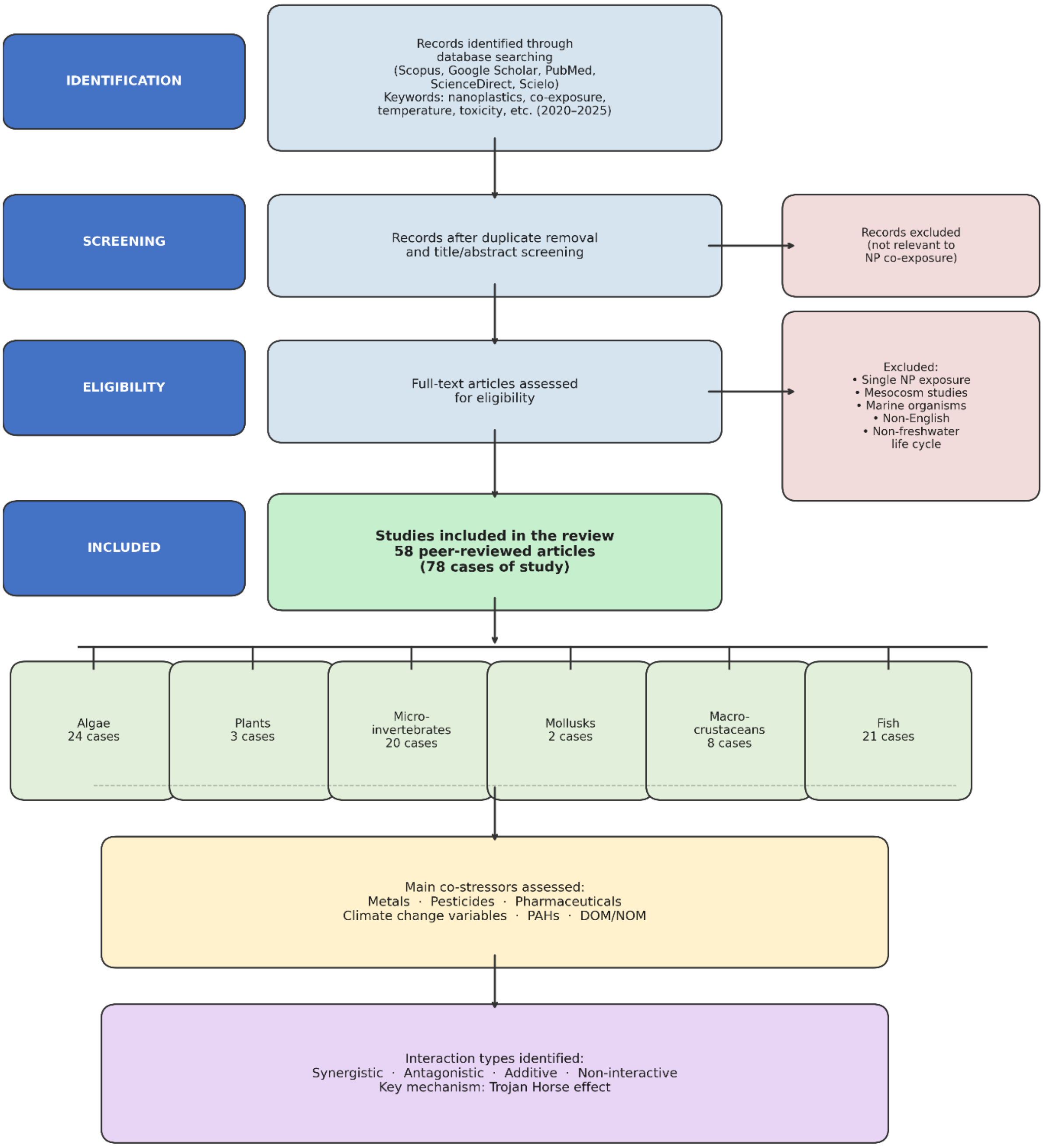

References

Arini, A., Muller, S., Coma, V., Grau, E., Sandre, O., Baudrimont, M., 2023. Origin, exposure routes and xenobiotics impart nanoplastics with toxic effects on freshwater bivalves. Environ. Sci. Nano 10, 1352–1371. https://doi.org/10.1039/d3en00022b

Banaee, M., Zeidi, A., Beitsayah, A., Multisanti, C.R., Faggio, C., 2025. Combined Effects of Nano-Polystyrene and Heavy Metal Mixture on the Bioaccumulation of Heavy Metals and Physiological Changes in Macrobrachium rosenbergii. J. Xenobiotics 15, 1–19. https://doi.org/10.3390/jox15040113

Bandeira, F.O., Alves, P.R.L., Hennig, T.B., Vaz, V.P., Vicentini, D.S., Juneau, P., Dewez, D., M, W.G., 2025. Individual and combined toxicity of polystyrene nanoplastics and clothianidin toward Daphnia magna, Lemna minor, Chlamydomonas reinhardtii, and Microcystis aeruginosa. Environ. Toxicol. Chem. 44, 470–483. https://doi.org/10.1093/etojnl/vgae029

Barreto, A., Silva, A.R.R., Capitão, A., Sousa, É.M.L., Calisto, V., Maria, V.L., 2023. Nanoplastics increase the toxicity of a pharmaceutical, at environmentally relevant concentrations – A mixture design with Daphnia magna. Environ. Toxicol. Pharmacol. 103. https://doi.org/10.1016/j.etap.2023.104258

Bottega, S., Fontanini, D., Ruffini Castiglione, M., Spanò, C., 2024. The impact of polystyrene nanoplastics on plants in the scenario of increasing temperatures: The case of Azolla filiculoides Lam. Plant Physiol. Biochem. 214. https://doi.org/10.1016/j.plaphy.2024.108946

Cao, J., Liao, Y., Yang, W., Jiang, X., Li, M., 2022. Enhanced microalgal toxicity due to polystyrene nanoplastics and cadmium co-exposure: from the perspective of physiological and metabolomic profiles. J. Hazard. Mater. 427. https://doi.org/10.1016/j.jhazmat.2021.127937

Chang, M., Li, M., Xu, W., Li, X., Liu, J., Stoks, R., Zhang, C., 2023. Microplastics increases the heat tolerance of Daphnia magna under global warming via hormetic effects. Ecotoxicol. Environ. Saf. 249, 114416. https://doi.org/10.1016/j.ecoenv.2022.114416

Che, S., Huang, M., Ma, H., Wan, Z., Feng, J., Ding, S., Li, X., 2024a. Toxic effects of nanopolystyrene and cadmium on the intestinal tract of the Chinese mitten crab (Eriocheir sinensis). Ecotoxicol. Environ. Saf. 270, 115936. https://doi.org/10.1016/j.ecoenv.2024.115936

Che, S., Huang, M., Zhu, L., Shen, L., Ma, Y., Wan, Z., Li, Xuguang, Zhou, J., Ding, S., Li,

Xilei, 2024b. Exposure to nanopolystyrene and phoxim at ambient concentrations causes oxidative stress and inflammation in the intestines of the Chinese mitten crab (Eriocheir sinensis). Ecotoxicol. Environ. Saf. 273, 116126. https://doi.org/10.1016/j.ecoenv.2024.116126

Christudoss, A.C., Chandrasekaran, N., Mukherjee, A., 2024. Polystyrene nanoplastics alter the ecotoxicological effects of diclofenac on freshwater microalgae Scenedesmus obliquus. Environ. Sci. Process. Impacts 26(1), 56–70. https://doi.org/10.1039/D3EM00341H

Das, S., Thiagarajan, V., Chandrasekaran, N., Ravindran, B., Mukherjee, A., 2022. Nanoplastics enhance the toxic effects of titanium dioxide nanoparticle in freshwater algae Scenedesmus obliquus. Comp. Biochem. Physiol. Part - C Toxicol. Pharmacol. 256, 109305. https://doi.org/10.1016/j.cbpc.2022.109305

Dayana, K., Rex M, C., Mukherjee, A., 2025. Combined toxic impact of polystyrene nanoplastics and a flame retardant tetrabromobisphenol A (TBBPA) on freshwater algae Scenedesmus obliquus. Environ. Pollut. Bioavailab. 37(1). https://doi.org/10.1080/26395940.2025.2575992

Ding, S., Che, S., Huang, M., Ma, Y., Shen, L., Feng, J., Li, X., 2025. Impacts of nanopolystyrene and/or phoxim exposure at environmentally relevant concentrations on the intestinal histopathology, intestinal microbiota, and metabolome in Eriocheir sinensis. Aquat. Toxicol. 278, 107168. https://doi.org/10.1016/j.aquatox.2024.107168

Duan, Z., Wang, J., Zhang, H., Wang, Y., Chen, Y., Cong, J., Gong, Z., Sun, H., Wang, L., 2023. Elevated temperature decreases cardiovascular toxicity of nanoplastics but adds to their lethality: A case study during zebrafish (Danio rerio) development. J. Hazard. Mater. 458, 131679. https://doi.org/10.1016/j.jhazmat.2023.131679

Fadare, O.O., Wan, B., Liu, K., Yang, Y., Zhao, L., Guo, L.H., 2020. Eco-Corona vs Protein Corona: Effects of Humic Substances on Corona Formation and Nanoplastic Particle Toxicity in Daphnia magna. Environ. Sci. Technol. 54, 8001–8009. https://doi.org/10.1021/acs.est.0c00615

Giri, S., Mukherjee, A., 2021. Ageing with algal EPS reduces the toxic effects of polystyrene nanoplastics in freshwater microalgae Scenedesmus obliquus. J. Environ. Chem. Eng. 9, 105978. https://doi.org/10.1016/j.jece.2021.105978

Guo, Y., O'Brien, A.M., Lins, T.F., Shahmohamadloo, R.S., Almirall, X.O., Rochman, C.M., Sinton, D., 2021. Effects of hydrogen peroxide on cyanobacterium Microcystis aeruginosa in the presence of nanoplastics. ACS ES&T Water 1(7), 1596–1607.

https://doi.org/10.1021/acsestwater.1c00090

Hanachi, P., Khoshnamvand, M., Walker, T.R., Hamidian, A.H., 2022. Nano-sized polystyrene plastics toxicity to microalgae Chlorella vulgaris: Toxicity mitigation using humic acid. Aquat. Toxicol. 245. https://doi.org/10.1016/j.aquatox.2022.106123

Huang, M., Ma, Y., Che, S., Shen, L., Wan, Z., Su, S., Ding, S., Li, X., 2024. Nanopolystyrene and phoxim pollution: A threat to hepatopancreas toxicity in Chinese mitten crab (Eriocheir sinensis). Aquat. Toxicol. 276, 107124. https://doi.org/10.1016/j.aquatox.2024.107124

Huang, P., Cao, L., Du, J., Guo, Y., Li, Q., Sun, Y., Zhu, H., Xu, G., Gao, J., 2025. Polystyrene nanoplastics amplify the toxic effects of PFOA on the Chinese mitten crab (Eriocheir sinensis). J. Hazard. Mater. 488, 137488. https://doi.org/10.1016/j.jhazmat.2025.137488

Iqbal, M., Siregar, M.N.F., Sofa, G.Z., Saputra, H.R., Pramudya, M., Dewi, F.R.P., Soegianto, A., Saputra, F., Aunurohim, Hayati, A., 2025. Nanoplastic-Induced Immune Modulation and Histopathological Changes in Oreochromis niloticus at Different Salinity Levels. J. Anim. Heal. Prod. 13, 235–242. https://doi.org/10.17582/journal.jahp/2025/13.2.235.242

Jia, H.B., Zhang, Y.H., Gao, R.Y., Liu, X.J., Shao, Q.Q., Hu, Y.W., Fu, L.M., Zhang, J.P., 2024. Combined Toxicity of Polystyrene Nanoplastics and Pyriproxyfen to Daphnia magna. Sustain. 16. https://doi.org/10.3390/su16104066

Kamel, W.A., Sayed, A.E.D.H., Idriss, S.K.A., Elbaghdady, H.A.M., 2025. Polyethylene nanoplastics intensify toxicity of potassium clavulanate in African catfish (Clarias gariepinus). Sci. Rep. 15, 1–17. https://doi.org/10.1038/s41598-025-27780-6

Khoshnamvand, M., You, D., Xie, Y., Feng, Y., Sultan, M., Pei, D.S., Fu, A., 2024. Alleviating binary toxicity of polystyrene nanoplastics and atrazine to Chlorella vulgaris through humic acid interaction: Long-term toxicity using environmentally relevant concentrations. Chemosphere 358. https://doi.org/10.1016/j.chemosphere.2024.142111

Lee, Y., Kim, D., Lee, J., Kim, H.S., 2024. Combined exposure to hypoxia and nanoplastics leads to negative synergistic oxidative stress-mediated effects in the water flea Daphnia magna. https://doi.org/10.1016/j.marpolbul.2024.116306

Li, F., Huang, J., Wei, X., Gong, Z., Xu, L., Gan, L., Chu, W., Yan, M., H., G., 2025. Antagonistic effect of polystyrene nanoplastics and sliver nanoparticles on Chlorella pyrenoidos. J. Environ. Sci. 161, 555–567. https://doi.org/10.1016/j.jes.2025.04.062

Li, H., Zhou, X., Huang, R., He, Q., Wu, Z., Huang, Y., Li, Z., Liu, Y., Xiaoliu, H., 2024. Combined toxicity of biochar with nanoplastics or silver nanoparticles toward Chlorella vulgaris. Algal Res. 78. https://doi.org/10.1016/j.algal.2024.103418

Lin, W., Jiang, R., Xiao, X., Wu, J., Wei, S., Liu, Y., Muir, D.C.G., Ouyang, G., 2020. Joint effect of nanoplastics and humic acid on the uptake of PAHs for Daphnia magna: A model study. J. Hazard. Mater. 391, 122195. https://doi.org/10.1016/j.jhazmat.2020.122195

Luo, B., Li, J., Wang, M., Zhang, X., Mi, Y., Xiang, J., Gong, S., Zhou, Y., Ma, T., 2022. Chronic toxicity effects of sediment-associated polystyrene nanoplastics alone and in combination with cadmium on a keystone benthic species Bellamya aeruginosa. J. Hazard. Mater. 433, 128800. https://doi.org/10.1016/j.jhazmat.2022.128800

Mamatimin, X., Song, W., Yilimulati, M., Zhang, T., Habibul, N., 2025. Impact of nanoplastics on the algicide efficacy of CuSO4 against Microcystis aeruginosa and the release of microcystin: A physiological and metabolomic analysis. J. Hazard. Mater. https://doi.org/10.1016/j.jhazmat.2025.138766

Maszczyk, P., Kiersztyn, B., Gozzo, S., Kowalczyk, G., Jimenez-Lamana, J., Szpunar, J., Pijanowska, J., Jines-Muñoz, C., Zebrowski, M.L., Babkiewicz, E., 2022. Combined Effects of Polystyrene Nanoplastics and Enrofloxacin on the Life Histories and Gut Microbiota of Daphnia magna. Water (Switzerland) 14. https://doi.org/10.3390/w14213403

Monikh, F.A., Vijver, M.G., Guo, Z., Zhang, P., Darbha, G.K., Peijnenburg, W.J.G.M., 2020. Metal sorption onto nanoscale plastic debris and trojan horse effects in Daphnia magna: Role of dissolved organic matter. Water Res. 186. https://doi.org/10.1016/j.watres.2020.116410

Nogueira, D.J., Silva, A.C. de O. da, da Silva, M.L.N., Vicentini, D.S., Matias, W.G., 2022. Individual and combined multigenerational effects induced by polystyrene nanoplastic and glyphosate in Daphnia magna (Strauss, 1820). Sci. Total Environ. 811. https://doi.org/10.1016/j.scitotenv.2021.151360

Oger, M.J.L., Bernay, B., Tessier, E., Amouroux, D., Kestemont, P., Cornet, V., 2025. The Trojan horse effect of nanoplastics exacerbates methylmercury-induced neurotoxicity during zebrafish development. Environ. Pollut. 384. https://doi.org/10.1016/j.envpol.2025.126966

Pashaei, R., Dzingelevičienė, R., Putna-Nimane, I., Overlinge, D., Błaszczyk, A., Walker, T.R., 2023. Acute toxicity of triclosan, caffeine, nanoplastics, microplastics, and their mixtures on Daphnia magna. Mar. Pollut. Bull. 192.

https://doi.org/10.1016/j.marpolbul.2023.115113
Saavedra, J., Stoll, S., Slaveykova, V.I. (2019)., 2019. Influence of nanoplastic surface charge on eco-corona formation, aggregation and toxicity to freshwater zooplankton. Environ. Pollut. 252, 715–722. https://doi.org/doi.org/10.1016/j.envpol.2019.05.135
Sanpradit, P., Byeon, E., Lee, Jin Sol, Jeong, H., Kim, H.S., Peerakietkhajorn, S., Lee, Jae Seong, 2024. Combined effects of nanoplastics and elevated temperature in the freshwater water flea Daphnia magna. J. Hazard. Mater. 465. https://doi.org/10.1016/j.jhazmat.2023.133325
Santos, A., Oliveira, M., Lopes, I., Almeida, M., Venâncio, C., 2024. Polyhydroxybutyrate (PHB) nanoparticles modulate metals toxicity in Hydra viridissima. Sci. Total Environ. 932. https://doi.org/10.1016/j.scitotenv.2024.172868
Şenol, O., Sulukan, E., Baran, A., Bolat, İ., Toraman, E., Alak, G., Yildirim, S., Bilgin, G., Ceyhun, S.B., 2023. Global warming and nanoplastic toxicity; small temperature increases can make gill and liver toxicity more dramatic, which affects fillet quality caused by polystyrene nanoplastics in the adult zebrafish model. Sci. Total Environ. 892. https://doi.org/10.1016/j.scitotenv.2023.164682
Soliman, A.M., Mohamed, A.S., Abdel-Khalek, A.A., Badran, S.R., 2025. Impact of polyvinyl chloride nano-plastics on the biochemical status of Oreochromis niloticus under a predicted global warming scenario. Sci. Rep. 15, 1–12. https://doi.org/10.1038/s41598-025-87558-8
Sulukan, E., Baran, A., Şenol, O., Yildirim, S., Mavi, A., Ceyhun, H.A., Toraman, E., Ceyhun, S.B., 2022. The synergic toxicity of temperature increases and nanopolystrene on zebrafish brain implies that global warming may worsen the current risk based on plastic debris. Sci. Total Environ. 808. https://doi.org/10.1016/j.scitotenv.2021.152092
Sun, Q., Yang, Y.T., Zheng, Z.Y., Ni, H.G., 2024. Nanopolystyrene size effect and its combined acute toxicity with halogenated PAHs on Daphnia magna. Sci. Total Environ. 912, 169435. https://doi.org/10.1016/j.scitotenv.2023.169435
Trevisan, R., Trimpey-Warhaftig, R., Gaston, K., Butron, L., Gaballah, S., Di Giulio, R.T., 2025. Polystyrene nanoplastics impact the bioenergetics of developing zebrafish and limit molecular and physiological adaptive responses to acute temperature stress. Sci. Total Environ. 958, 178026. https://doi.org/10.1016/j.scitotenv.2024.178026
Wan, J.K., Chu, W.L., Kok, Y.Y., Lee, C.S., 2021. Influence of polystyrene microplastic and nanoplastic on copper toxicity in two freshwater microalgae. Environ. Sci. Pollut. Res. 28(25), 33649–33668. https://doi.org/10.1007/s11356-021-12983-x

Wang, F., Wang, B., Qu, H., Zhao, W., Duan, L., Zhang, Y., Zhou, Y., Yu, G., 2020. The influence of nanoplastics on the toxic effects, bioaccumulation, biodegradation and enantioselectivity of ibuprofen in freshwater algae Chlorella pyrenoidosa. Environ. Pollut. 263. https://doi.org/10.1016/j.envpol.2020.114593

Wang, Q., Wang, J., Chen, H., Zhang, Y., 2023. Toxicity effects of microplastics and nanoplastics with cadmium on the alga Microcystis aeruginosa. Environ. Sci. Pollut. Res. 30(7), 17360–17373. https://doi.org/10.1007/s11356-022-23278-0

Wang, Z., Qin, L., Li, Z., Liu, M., Hu, X., Yin, D., 2024. The combined effects of polystyrene nanoplastics and dissolved organic matter on the environmental bioavailability of carbamazepine. J. Hazard. Mater. 480, 136031. https://doi.org/10.1016/j.jhazmat.2024.136031

Xin, X., Chen, B., Péquin, B., Song, P., Yang, M., Song, X., Zhang, B., 2022. Binary toxicity of polystyrene nanoplastics and polybrominated diphenyl ethers to Arctic Cyanobacteria under ambient and future climates. Water Res. 226. https://doi.org/10.1016/j.watres.2022.119188

Xu, J., Feng, G., Yan, Y., 2025a. Effects of polystyrene nanoplastics and copper on gill tissue structure, metabolism, and immune function of the Chinese mitten crab (Eriocheir sinensis). Front. Mar. Sci. 12, 1–16. https://doi.org/10.3389/fmars.2025.1538734

Xu, J., Feng, G., Yan, Y., 2025b. New insights into the responding mechanism of Eriocheir sinensis hepatopancreas under nanoplastics and copper stress by transcriptome analysis. J. Environ. Manage. 393, 126792. https://doi.org/10.1016/j.jenvman.2025.126792

Xu, W., Chang, M., Li, J., Li, M., Stoks, R., Zhang, C., 2024. Local thermal adaption mediates the sensitivity of Daphnia magna to nanoplastics under global warming scenarios. J. Hazard. Mater. 476, 134921. https://doi.org/10.1016/j.jhazmat.2024.134921

Yan, Z., Zhou, Y., Zhu, P., Bao, X., Su, P., 2023. Polystyrene nanoplastics mediated the toxicity of silver nanoparticles in zebrafish embryos. Front. Mar. Sci. 10, 1–11. https://doi.org/10.3389/fmars.2023.1195125

Yang, J., Yang, Z., Wang, J., Liang, Y., Zeng, H., Qin, L., Song, X., Mo, L., 2025. Toxic effects and mechanisms of nanoplastics and sulfonamide antibiotics on Scenedesmus obliquus. Ecotoxicol. Environ. Saf. 289, 117685. https://doi.org/10.1016/j.ecoenv.2025.117685

Yang, Y., Guo, Y., O'Brien, A.M., Lins, T.F., Rochman, C.M., Sinton, D., 2020. Biological

Responses to Climate Change and Nanoplastics Are Altered in Concert: Full-Factor Screening Reveals Effects of Multiple Stressors on Primary Producers. Environ. Sci. Technol. 54, 2401–2410. https://doi.org/10.1021/acs.est.9b07040

Yu, G., Huang, S., Luo, X., Zhao, W., Zheng, Z., 2022. Single and combined toxicity effects of nanoplastics and bisphenol F on submerged the macrophyte Hydrilla verticillata. Sci. Total Environ. 814, 152564. https://doi.org/10.1016/j.scitotenv.2021.152564

Zhang, S., Sun, Z., Zheng, T., He, C., Lin, D., 2024. Nanoplastics increase algal absorption and toxicity of Cd through alterations in cell wall structure and composition. Water Res. 254. https://doi.org/10.1016/j.watres.2024.121394

Zhang, Y., Goss, G.G., 2021. The “trojan Horse” effect of nanoplastics: Potentiation of polycyclic aromatic hydrocarbon uptake in rainbow trout and the mitigating effects of natural organic matter. Environ. Sci. Nano 8, 3685–3698. https://doi.org/10.1039/d1en00738f

Zhang, Y.H., Gao, R.Y., Zhu, F., Fu, L.M., Zhang, J.P., 2024. Combined effect of polystyrene nanoparticles and chlorpyrifos to Daphnia magna. Chemosphere 369, 143765. https://doi.org/10.1016/j.chemosphere.2024.143765
